# Age-related proteostasis and metabolic alterations in *Caspase-2*-deficient mice

**DOI:** 10.1038/cddis.2014.567

**Published:** 2015-01-22

**Authors:** C H Wilson, S Shalini, A Filipovska, T R Richman, S Davies, S D Martin, S L McGee, J Puccini, A Nikolic, L Dorstyn, S Kumar

**Affiliations:** 1 Centre for Cancer Biology, University of South Australia, Adelaide, South Australia, Australia; 2 Mitochondrial Medicine and Biology Group, Harry Perkins Institute of Medical Research, Nedlands, Western Australia, Australia; 3 School of Chemistry and Biochemistry, The University of Western Australia, Nedlands, Western Australia, Australia; 4 Metabolic Remodelling Laboratory, School of Medicine, Deakin University, Geelong, Victoria, Australia; 5 Metabolism and Inflammation Program, Baker IDI Heart and Diabetes Institute, Melbourne, Victoria, Australia

## Abstract

Ageing is a complex biological process for which underlying biochemical changes are still largely unknown. We performed comparative profiling of the cellular proteome and metabolome to understand the molecular basis of ageing in *Caspase-2-*deficient (*Casp2*^*−/−*^) mice that are a model of premature ageing in the absence of overt disease. Age-related changes were determined in the liver and serum of young (6–9 week) and aged (18–24 month) wild-type and *Casp2*^*−/−*^ mice. We identified perturbed metabolic pathways, decreased levels of ribosomal and respiratory complex proteins and altered mitochondrial function that contribute to premature ageing in the *Casp2*^*−/−*^ mice. We show that the metabolic profile changes in the young *Casp2*^*−/−*^ mice resemble those found in aged wild-type mice. Intriguingly, aged *Casp2*^*−/−*^ mice were found to have reduced blood glucose and improved glucose tolerance. These results demonstrate an important role for caspase-2 in regulating proteome and metabolome remodelling during ageing.

Ageing is a complex biological process involving the accumulation of cellular damage and degeneration of repair systems over time resulting in perturbed homeostasis, physiological decline, age-related disease and death. Age-related deterioration of metabolic, inflammatory, cardiovascular and neurological systems contributes to the development of pathologies, such as obesity, type II diabetes, Alzheimer's, Parkinsons and cancer.^1^ Unifying features of ageing and its related pathologies include perturbed stress response pathways, increased oxidative stress induced damage and disruption of metabolic and energy systems homeostasis.^2^ Although the mechanistic driving force behind age-related metabolic reprogramming is still unknown, mitochondrial dysfunction and de-regulated nutrient sensing are two contributing features that have been proposed to be the hallmarks of ageing.^3^ Furthermore, many of the evolutionary conserved genes and pathways associated with longevity have multifunctional roles in metabolism.^4, 5^ Proteomic and metabolite profiling are valuable methods to enable characterization of the system-wide molecular changes during ageing.

Caspase-2 (Casp2) is the most evolutionarily conserved member of the caspase family of proteases, known for their roles in apoptosis and inflammatory responses.^6, 7^ Casp2 has been shown to have both apoptotic and non-apoptotic functions in stress response pathways, maintaining genomic integrity, tumour suppression and ageing.^6, 7, 8, 9, 10, 11^ In the presence of oncogenic stress, *Casp2* deficiency in mice results in enhanced cellular transformation, genomic instability and increased tumorigenesis.^9, 10, 12^ *Casp2*-deficient (*Casp2*^*−/−*^) mice exhibit subtle phenotypic changes, including premature ageing-related traits, impaired oxidative stress defence and increased oxidative tissue damage.^11, 13^ We previously found the impaired antioxidant response to be partly due to decreased expression of stress response transcription factors FoxO1 and FoxO3a.^11^ However, the mechanism by which Casp2 regulates these factors and its pathophysiological role in ageing is still unknown. Furthermore, none of the known defined substrates of Casp2 appear to contribute to its ageing role.^11^

In apoptosis, Casp2 activation occurs in response to a wide-range of stress-induced stimuli (reviewed in Kumar^6^) and altered metabolic flux.^14^ Studies in *Xenopus oocytes* and mammalian cells provide evidence that Casp2 is metabolically regulated, acting as a sensor to changes in NADPH levels resulting from altered flux through the pentose-phosphate pathway (PPP).^14, 15^ Casp2 has also been implicated in lipid metabolism. Phenotypically, *Casp2*^*−/−*^ mice have reduced maximal body weight, reduced body fat content and significantly reduced levels of subcutaneous adipose tissue^11, 13^ and are protected from diabetes-induced marrow adiposity.^16^ In addition, rat *Casp2* increases in response to high-fat diet,^17, 18^ and human *CASP2* is transcriptionally regulated by the sterol regulatory element binding proteins (SREBPs).^19^ Recently, it was suggested that Casp2 can initiate lipid induced apoptosis (lipoapoptosis) caused by saturated fatty acid-induced lipotoxicity.^20, 21^

The mechanism by which loss of Casp2 alters key metabolic pathways and regulates the oxidative stress response and ageing is currently unknown. The premature ageing phenotype in *Casp2*^*−/−*^ mice, which occurs in the absence of any concomitant age-related disease, provides us with a unique model in which to carry out system-wide investigations to enhance our understanding of Casp2 in metabolic disorders and the biology of ageing.

Here we used comparative proteomics and metabolomics to analyse ageing in wild-type (WT) and *Casp2*^*−/−*^ mice. Given the central role that the liver has in organismal metabolic homeostasis and detoxification, we focussed on profiling the proteome and metabolome in the liver and changes in serum metabolites during ageing. Our data provide unique molecular insight into the pathways underlying the changes that occur during ageing such as decreased oxidative phosphorylation (OXPHOS) and ribosomal proteins and the role of Casp2 in the regulation of age-related metabolic reprogramming, mitochondria function and glucose tolerance.

## Results

### Global analysis of protein and metabolite changes in the liver and serum during ageing

**Figure 1:**
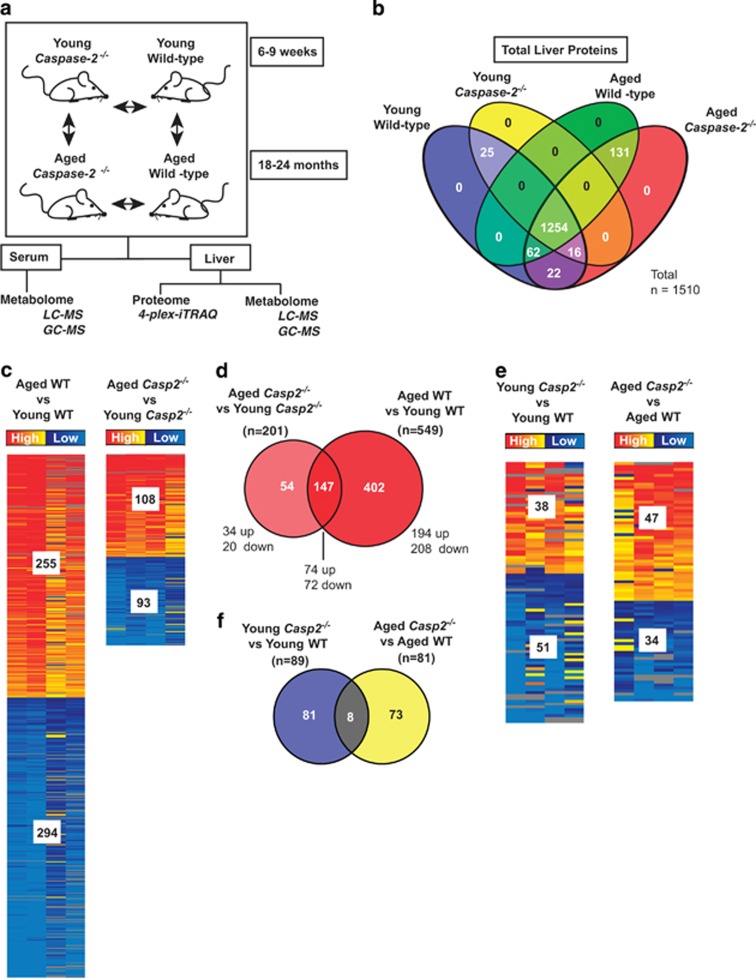
Experimental design and proteome profile of the liver during ageing of WT and *Casp2*^*−/−*^ mice. (**a**) Experimental design for proteomics analysis of liver tissue and metabolomics analysis of the serum and liver of young (6–9 weeks) and aged (18–24 months) WT and *Casp2*^*−/−*^ mice. (**b**) Four-way Venn diagram summary of the total number of unique and overlapping high confidence liver proteins identified by iTRAQ analysis in each of the four groups (*n*=4/group). (**c**) Heat maps of liver proteins differentially expressed during ageing of WT (left panel, Aged WT *versus* Young WT) and *Casp2*^*−/−*^ mice (right panel, Aged *Casp2*^*−/−*^ *versus* Young *Casp2*^*−/−*^). (**d**) Two-way Venn diagram summary of unique and overlapping liver proteins that are differentially expressed during ageing of WT (Aged WT *versus* Young WT) and *Casp2*^*−/−*^ (Aged *Casp2*^*−/−*^ *versus* Young *Casp2*^*−/−*^) mice. (**e**) Heat maps of liver proteins differentially expressed between *Casp2*^*−/−*^ and WT mice in young (left panel, Young *Casp2*^*−/−*^ *versus* Young WT) and aged (Aged *Casp2*^*−/−*^ *versus* Aged WT) mice. (**f**) Two-way Venn diagram summary of unique and overlapping liver proteins that are differentially expressed in young and aged *Casp2*^*−/−*^ mice relative to WT mice. Heat maps display the number of increased (red) or decreased (blue) proteins. Heat map columns represent the abundance of proteins in individual mice relative to average of the comparison group. For a complete list of unique and overlapping proteins in each group, see output from Venn diagram in Supplementary Table S1. For a complete list of differentially abundant proteins, see Supplementary Table S2. See Supplementary Figure S1

We performed omics analysis of the liver and serum from young (6–9 week) and aged (18–24 month), WT and *Casp2*^*−/−*^ mice to understand the molecular basis of ageing (Figure 1a). Liver proteomes (*n*=4/group) were analysed in six 4-plex iTRAQ experiments using a pooled internal standard in each run. Metabolomic analyses of the livers (*n*=7–8/group) and sera (*n*=6/group) were performed by untargeted GC-MS analysis of polar metabolites, targeted GC-MS analyses of organic and fatty acids and LC-MS quantitation of amine metabolites. Biochemical analysis of liver functional enzymes in the sera revealed no differences in their activities between WT and *Casp2*^*−/−*^ mice.

In total, 1510 proteins were identified with high confidence, from two or more peptides, among the four groups by iTRAQ analysis (Figure 1b). Most proteins (1254, 83%) were mutually identified in each group, while 25 (1.7%) and 131 (8.7%) proteins were uniquely identified in young and aged mice, respectively (Figure 1b,Supplementary Table S1). No proteins were unique to either genotype. There was no significant difference in abundance of the proteins identified exclusively in each age group between genotypes (Supplementary Tables S1 and S2).

Differentially abundant proteins of interest were identified as being those with *P*<0.05 or those with a ratio ≥1.2 or ≤0.83 and *P*<0.1. During ageing of WT mice, the abundance of 549 (255 up and 294 down; 36%) proteins changed. In contrast, abundance of only 201 proteins (108 up and 93 down; over 60% less proteins that are altered during ageing of WT mice) were altered during ageing of *Casp2*^*−/−*^ mice (Figures 1c and d and Supplementary Table S2). This suggests that normal age-related changes in protein abundance are affected in *Casp2*^*−/−*^ mice. Further analysis revealed that the majority of these proteins do undergo similar, but non-significant, changes during ageing of *Casp2*^*−/−*^ mice compared with WT (Supplementary Table S2). Most proteins altered during ageing of both genotypes (147, 73%) have similar changes in abundance, thus likely occur independent of Casp2 (Figure 1d). Some of these changes are consistent with other proteomics studies of the ageing liver such as increased epoxide hydrolase 2, 3-ketoacyl-CoA thiolase and decreased NADH dehydrogenase (ubiquinone) iron–sulfur protein 8 (NDUFS8).^22, 23, 24^ Some proteins that change with age in WT but not in *Casp2*^*−/−*^ mice include increases in a number of glucose-metabolising enzymes, including fructose-1,6-bisphophatase, triosephosphate isomerase and phosphoglycerate kinase 1 and decreases in a substantial number (>40) of 40S and 60S ribosomal proteins (Supplementary Table S2).

Interestingly, for >30 of the proteins not altered during ageing of *Casp2*^*−/−*^ mice, their abundance in young *Casp2*^*−/−*^ mice was found to be characteristic of those found in aged WT (Supplementary Table S2), for example, increased abundance of glucose-metabolising triosephosphate isomerase, amino-acid-metabolising cystathionine beta-synthase and glycine *N*-methyltransferase and decreased abundance of detoxification enzymes UDP-glucuronosyltransferase 2B17, cytochrome P450 (CYP) 2C54, CYP2C50, CYP2A12 and CYP2C40.

Within each age group, loss of Casp2 resulted in altered abundance of approximately 5% of the total proteins with 89 and 81 proteins changing in young and aged *Casp2*^*−/−*^ compared with WT mice (Figures 1e and f and Supplementary Figure S1A). Of these, only eight were commonly altered between genotypes within each age group (Figure 1f). This is likely due to the vast changes in protein expression that occur during ageing and suggests that a small subset of proteins are affected by loss of Casp2.

Age-related changes in protein expression contribute to some of the significant differences observed in the expression of proteins between aged *Casp2*^*−/−*^ and WT mice, for example, mitochondrial L-2 hydroxyglutarate dehydrogenase, mitochondrial sulphide:quinone oxidoreductase and calpastatin (Supplementary Figure S1B and Supplementary Table S2).

### Metabolomics analysis

**Figure 2:**
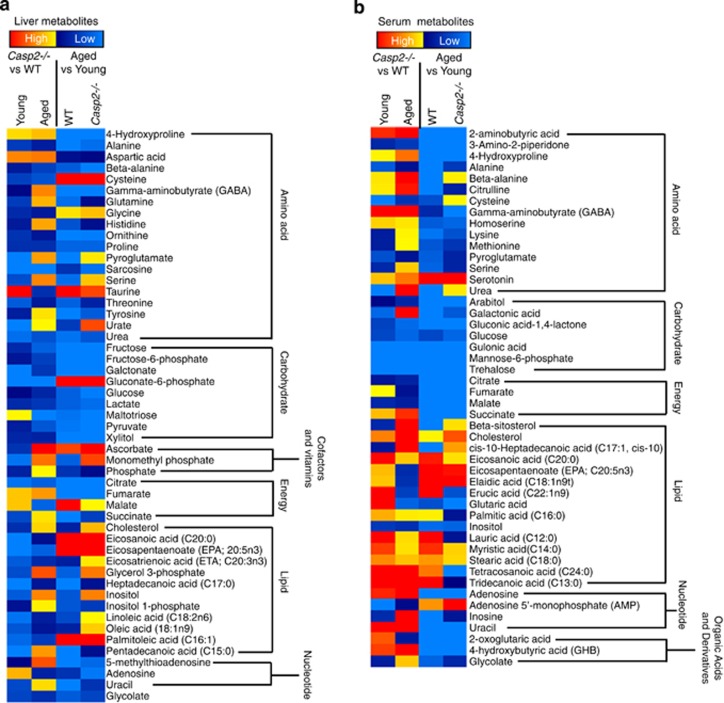

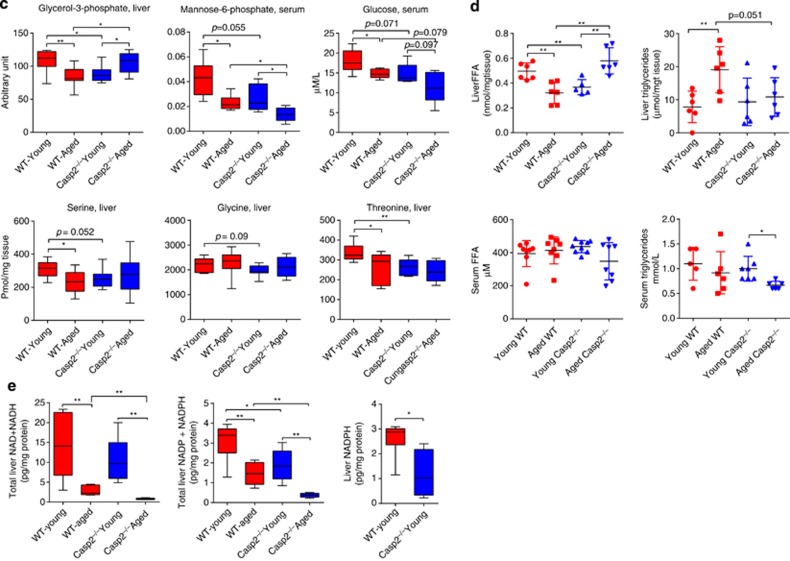
Differential liver and serum metabolome profiles of WT and *Casp2*^*−/−*^ mice during ageing. (**a** and **b**) Heat maps of metabolites that are differentially altered in the (**a**) liver and (**b**) serum during ageing of WT and *Casp2*^*−/−*^ mice and between genotypes within each age group. Heat map columns represent the average abundance of metabolites (*n*=6–8/group). (**c**) Example of some liver and serum metabolites that are significantly altered in abundance. (**d**) FFA and triglyceride levels in the liver and serum of young and aged WT and *Casp2*^*−/−*^ mice. (**e**) Pyridine nucleotide (NAD^+^, NADH, NADP^+^, NADPH) levels in young and aged WT and *Casp2*^*−/−*^ mice. Red-coloured boxes represented WT samples, and blue-coloured boxes represent *Casp2*^*−/−*^ samples. Values are expressed as means±S.D. or as box-and-whisker plots, with values on top and at the bottom of the box representing 75th and 25th percentile and bar ‘whiskers' indicating the 10th and 90th percentile. *P<0.05, **P<0.01 (*n*=6–8/group). Individual values can be found in Supplementary Table S3. See Supplementary Figure S2

Metabolomic analyses identified a total of 110 (91 known) and 133 (113 known) metabolites in the liver and serum, respectively, of young and aged WT and *Casp2*^*−/−*^ mice (Supplementary Table S3). Metabolites with a significant (*P*<0.05) difference between groups or a trend in altered levels (*P*<0.10) were selected as metabolites of interest (Supplementary Table S3). In total, all comparisons revealed differential abundance of 51 and 48 known metabolites in the liver and serum, respectively (Figures 2a and b, Supplementary Figure S2 and Supplementary Table S3). Of these, 35/91 (38%) and 27/91 (30%) liver metabolites and 38/113 (34%) and 19/113 (17%) serum metabolites were altered in WT and *Casp2*^*−/−*^, respectively (Figures 2a and b, and Supplementary Figure S2). In the serum, a 50% reduction in the number of age-related changes in metabolite abundance was observed in *Casp2*^*−/−*^ mice relative to WT mice, consistent with observations in the liver proteome (Figure 2b).

Ageing was associated with decreased amino acid and carbohydrate metabolites and altered energy and lipid metabolism in both *Casp2*^*−/−*^ and WT mice (Figures 2a and b,Supplementary Figure S2, and Supplementary Table S3). Altered levels of several of these metabolites have been previously associated with ageing, including alanine, serine, methionine, lactate, glycerol-3 phosphate (G3P), glucose, fructose-6-phosphate, citrate and some fatty acids.^25, 26, 27, 28^ Most metabolites decreased in abundance during ageing, with 31/35 (89%) and 20/27 (74%) liver metabolites and 31/38 (82%) and 16/19 (84%) serum metabolites changing in WT and *Casp2*^*−/−*^ mice respectively (Figures 2a and b, Supplementary Figures S2C, D, G and H, and Supplementary Table S3). Most metabolites that increased with age were fatty acids in WT serum of which many were saturated fatty acids (Figure 2b and Supplementary Figure S2G). Interestingly, in common with aged WT mice, increased fatty acids were detected in the serum of young *Casp2*^*−/−*^ mice, including saturated fatty acids palmitic acid (C16:0), myristic acid (C14:0), stearic acid (C18:0) and tridecanoic acid (C13:0) (Figure 2b and Supplementary Figure S2F). Several other metabolites had abundant levels in young *Casp2*^*−/−*^ mice that were characteristic of aged WT mice, including decreased urea, uracil, pyroglutamate, serine, threonine, inositol-1-phosphate and G3P in the liver (Figures 2a–d and Supplementary Figure S2).

G3P, an intermediate of glycolysis and lipid metabolism, was significantly altered during ageing of both genotypes (decreased in WT, increased in *Casp2*^*−/−*^) and in both young and aged *Casp2*^*−/−*^ mice relative to WT (Figure 2c,Supplementary Figure S2 and Supplementary Table S3). In serum, only two metabolites, glucose and mannose-6-phosphate, were altered across all comparisons, including lower abundance of both metabolites in young *Casp2*^*−/−*^ relative to WT (Figure 2c,Supplementary Figure S2 and Supplementary Table S3). This suggested that glucose homeostasis may be altered in *Casp2*^*−/−*^ mice. An age-associated decline in liver glucose was also observed in WT and *Casp2*^*−/−*^ mice but not between genotypes (Supplementary Figures S2C and D).

Ageing resulted in the abundance of 17 metabolites being significantly altered in both the liver and serum. In particular, 4-hydroxyproline was the metabolite most affected with age in both genotypes and tissues being reduced more than 3-fold (*P<*0.001) in the liver and 2-fold (*P*<0.0001) in the serum (Supplementary Table S3). Interestingly, the fatty acid omega-3 (eicosapentaenoic acid, EPA; C20:5n3) increased in both the liver and serum of WT and *Casp2*^*−/−*^ mice during ageing, while metabolites associated with energy homeostasis (citrate, fumarate, succinate) and carbohydrate metabolism (glucose, gulonic acid, mannose-6-phosphate, trehalose) decreased (Supplementary Figures S2C and D).

Liver metabolites most significantly (*P*<0.01 or *P*<0.001) altered by the loss of Casp2 were urea, threonine, inositol-1-phopshate and galactonate and serum metabolites were beta-alanine, cysteine and the short-chain fatty acid, tridecanoic acid (C13:0) (Supplementary Figure S2 and Supplementary Table S3).

Total free fatty acids (FFAs) and lipids were also measured. In the liver, the pattern of FFA levels were similar to G3P where FFA was significantly decreased with age in WT mice while it was increased in *Casp2*^*−/−*^ mice, and similar to aged WT, FFA was significantly lowered in young *Casp2*^*−/−*^ mice (Figure 2d). No significant difference was observed in serum FFA. Total triglycerides increased with age in the WT liver but not in the serum (Figure 2d). Liver triglycerides did not change with age in the *Casp2*^*−/−*^ liver; however, a small but significant decrease was observed in serum (Figure 2d).

Important for metabolic flux, pyridine nucleotides (NAD, NADH, NAD, NADPH), were measured in the liver. An age-related decline in the level of total pyridine nucleotides was observed, with a significant reduction in the aged *Casp2*^*−/−*^ *versus* WT livers (Figure 2e). Interestingly, in young mice the level of NADPH was significantly reduced in *Casp2*^*−/−*^ animals (Figure 2e). There were no significant differences in NAD/NADH (nicotinamde-adenine dinucleotide) or NADP/NADPH (nicotinamide-adenine dinucleotide phosphate) ratios.

### Biological function and expression pathway analyses of ageing

Differentially abundant proteins were functionally characterized by performing enrichment analysis of biological processes and pathways using DAVID Bioinformatics Resources 6.7 (http://david.abcc.ncifcrf.gov/). Ageing mostly affected metabolic pathways, including increases in carbohydrate, amino acid and fatty acid metabolism and decreases in electron transport, OXPHOS and protein biosynthesis (Supplementary Figures S3A and B and Supplementary Table S4). Biological processes enhanced in young *Casp2*^*−/−*^ compared with WT mice included amino-acid metabolism while, in common with ageing, processes with reduced expression of proteins were mostly associated with steroid metabolism, electron transport and fatty acid metabolism (Supplementary Figure S3C and Supplementary Table S4). Interestingly, electron transport was the most increased biological process in aged *Casp2*^*−/−*^ *versus* WT mice (Supplementary Figure S3D and Supplementary Table S4).

**Figure 3:**
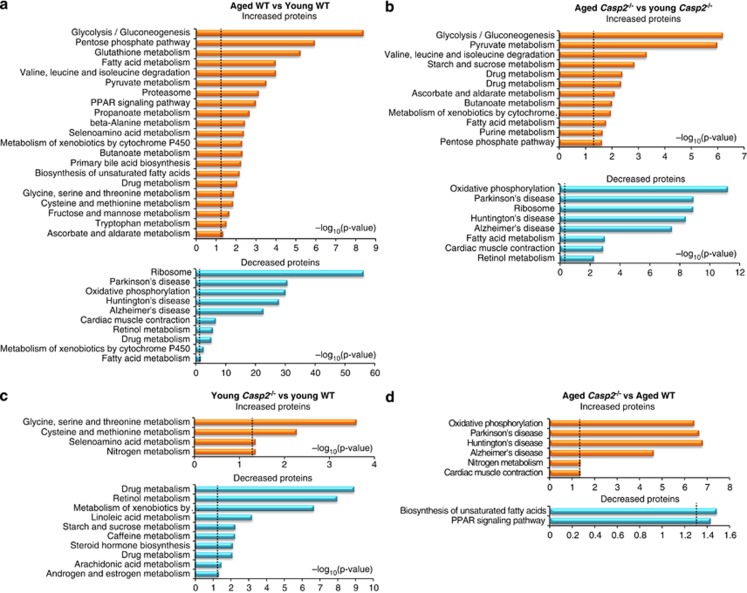
Metabolic and mitochondrial pathways are altered in the liver during ageing of WT and *Casp2*^*−/−*^ mice. Pathway enrichment analysis of liver proteins that are differentially abundant during ageing of (**a**) WT mice, (**b**) *Casp2*^*−/−*^ mice and between genotypes of (**c**) young and (**d**) aged *Casp2*^*−/−*^ and WT mice. Orange colour represents increased proteins, and blue represents decreased proteins.Vertical lines display cutoff for significance of (−log10(*P*-value)) with Benjamini's correction. Enrichment of Kyoto Encyclopedia of Genes and Genomes (KEGG) pathway was performed using DAVID Bioinformatics resources 6.7 (http://david.abcc.ncifcrf.gov). Details of individual proteins included in each enriched biochemical pahtway can be found in Supplementary Table S5. See Supplementary Figure S3

Pathway enrichment analysis revealed OXPHOS, ribosome, glycolysis/gluconeogenesis, drug (via CYPs) and fatty acid metabolism to be some of the most significant (Benjamini corrected *P*<0.05) biochemical pathways affected with age (Supplementary Figures S3E and F and Supplementary Table S5). Although pathways for Parkinson's, Huntington's and Alzheimer's disease were highly significant, this enrichment was most likely due to the majority of proteins in these pathways overlapping with the OXPHOS pathway. Glycolysis/gluconeogenesis, pyruvate metabolism, valine, leucine and isoleucine degradation increased during ageing of WT and *Casp2*^*−/−*^ mice, whereas OXPHOS and ribosome pathways decreased (Figures 3a and b and Supplementary Table S5). Fatty acid metabolism was significantly enriched in both increased and decreased protein sets during ageing of both genotypes as was drug metabolism in ageing WT only (Figures 3a and b and Supplementary Table S5). Downregulation of pathways involved in fatty acid metabolism and xenobiotic/drug metabolism by CYP during ageing are consistent with previous findings.^29^

Some pathways of amino-acid metabolism were only altered (mostly increased) during ageing of WT mice (Figures 3a and b and Supplementary Table S5). In young *Casp2*^*−/−*^ mice, an increase in three of these pathways, including selenocysteine, glycine, serine and threonine metabolism and cysteine and methionine metabolism, was observed (Figure 3c and Supplementary Table S5). In addition, young *Casp2*^*−/−*^ mice exhibited significant decreases in drug metabolism, retinol metabolism, metabolism of xenobiotics by CYP's and linoleic acid metabolism pathways similar to that observed during ageing of WT mice (Figure 3c).

Similar to protein analyses, metabolite set-enrichment was carried out using Metaboanalyst 2.0 (http://www.metaboanalyst.ca). Comparison of pathways enriched by metabolite sets in the liver reveals overlap with proteomic pathways, including age-related changes to the citric acid cycle and mitochondrial electron transport chain and altered glycine, serine and threonine metabolism in young *Casp2*^*−/−*^ (Supplementary Figure S4). Age-related changes to the citric acid cycle are also reflected in serum metabolomics data (Supplementary Figure S5).

### Casp2 deficiency alters the levels of amino-acid- and lipid-metabolising enzymes

**Figure 4:**
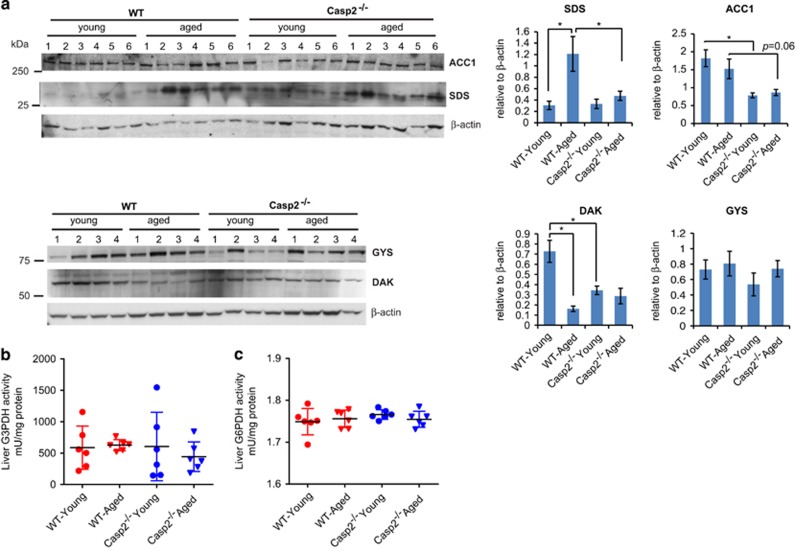
Validation of proteomics and metabolomics findings. Validation of proteomics findings in the liver lysates from young and aged WT and *Casp2*^*−/−*^ mice. (**a**) Western blotting of ACC1, serine/threonine-degrading enzyme (SDS), DAK and GYS. Histograms display quantitation of the protein levels relative to *β*-actin loading control. (**b** and **c**) Liver enzymatic of (**b**) glycerol-3-phosphate dehydrogenase (G3PDH) and (**c**) glycerol-6-phosphate dehydrogenase (G6PDH) in liver extracts. (**a**) Values are means±S.E.M. (**b** and **c**) Values are means±S.D. *P<0.05; **P<0.01 (*n*=4–6/group)

The levels of G3P, NADPH, FFA and amino acids glycine, serine and threonine were altered during ageing of WT mice and as a result of *Casp2* deficiency. As G3P is a metabolite common to carbohydrate, amino acid and lipid metabolism, it is likely that changes in these pathways are interconnected. Immunoblotting was performed to validate differences in enzymes from these pathways that were observed by proteomics to be different in the *Casp2*^*−/−*^ mice. Although proteomic analyses indicated increased levels of the serine/threonine-degrading enzyme (L-serine dehydratase/L-threonine deaminase (SDS)) (ratio 1.27, *P*=0.06) in the livers of young *Casp2*^*−/−*^ mice, immunoblotting revealed a significant increase of SDS with age in WT, and to a lesser degree in the *Casp2*^*−/−*^ mice (Figure 4a). Increased SDS activity has been previously observed with age.^30^ The enzyme biofunctional ATP-dependent dihydroxyacetone kinase/FAD-AMP lyase (cyclizing) (DAK), an important mediator in the production of the G3P precursor dihydroxyacetone phosphate (DHAP), was identified by proteomics to be significantly decreased during ageing of WT (ratio 0.84, *P*=0.023) and in young *Casp2*^*−/−*^ *versus* WT mice (ratio 0.85, *P*=0.043). This was confirmed by immunoblotting (Figure 4a). Proteomics and biochemical assays did not reveal any differences in levels or activity of another primary G3P-metabolizing enzyme, G3P dehydrogenase G3PDH (Figure 4b). The decreased abundance of acetyl-CoA carboxylase 1 (ACC1) protein, an important enzyme involved in *de novo* fatty acid synthesis, in the young *Casp2*^*−/−*^ liver (ratio 0.82, *P*=0.03) was confirmed by immunoblotting (Figure 4a).

### Relationship between low NADPH levels and the PPP in *Casp2*^*−/−*^ mice

NADPH is primarily produced through the PPP. To investigate why NADPH levels may be decreased in young *Casp2*^*−/−*^, we assessed the enzymatic activity of one of the primary PPP enzymes generating NADPH, glucose-6-phosphate dehydrogenase (G6PDH); however, there was no significant difference between the groups (Figure 4c).

Altered glycogen metabolism and accumulation can lead to increased reactive oxygen species (ROS) and alter flux through the PPP.^31^ Proteomics revealed significant decrease in glycogen phosphorylase (PYGL) in young *Casp2*^*−/−*^ *versus* WT (ratio 0.75, *P*=0.048) and increases with age in *Casp2*^*−/−*^ (ratio 1.42, *P*=0.053). Glycogen synthase (GYS) decreased with age in WT (ratio 0.082, *P*=0.085) and in young *Casp2*^*−/−*^ *versus* WT (ratio 0.76, *P*=0.029) mice. Immunoblotting confirmed a trend towards decreased GYS protein in young *Casp2*^*−/−*^ mice (Figure 4a). Levels of PYGL and GYS normally change in opposite direction to each other. The decreased abundance of both enzymes is suggestive of an adaptive response that would unlikely contribute to altered flux of glycogen metabolites through the PPP and thus the low NADPH levels observed in *Casp2*^*−/−*^ mice.

### Casp2 contributes to altered mitochondrial function during ageing

**Figure 5:**
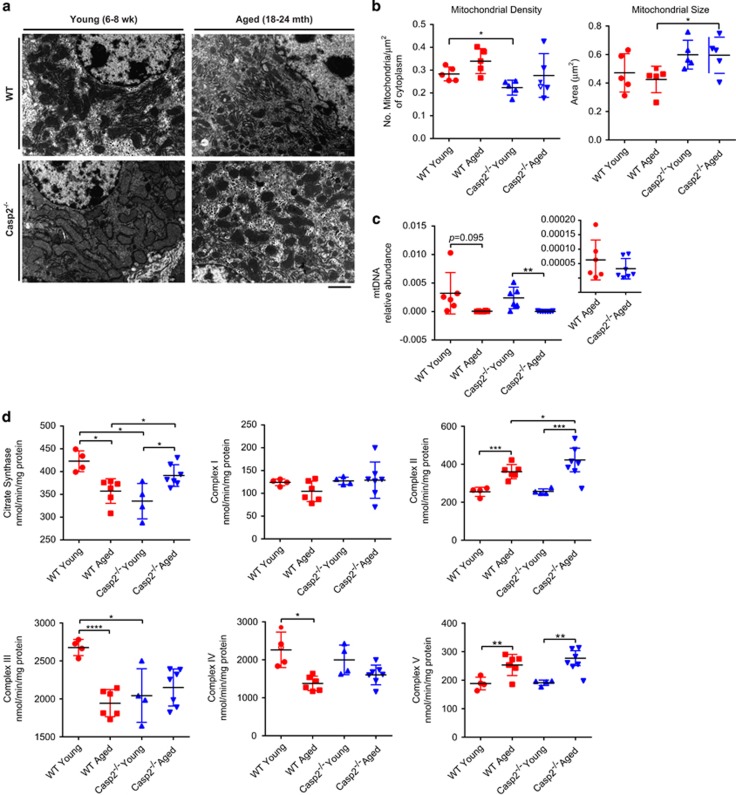
Altered mitochondria and OXPHOS in the livers of *Casp2*^*−/−*^ mice contributes to ageing. (**a**) Representative electron microscopic images of hepatocyte mitochondria in the liver sections. Images representatives of 3–5 fields of view for each individual sample. (**b**) Hepatocyte mitochondria density (no. of mitochondria/*μ*m^2^ of cytosplasm) measured from images of 3–5 fields of view for each individual and mitochondria size measured from >80 mitochondria across 3–5 fields of view for each individual (*n*=5/group). (**c**) Mitochondrial DNA (mtDNA) content as measured by qPCR. (**d**) Mitochondrial citrate synthase and OXPHOS complex enzyme activity in isolated mitochondria. Values are means±S.D. *P<0.05; **P<0.01; ***P<0.001, ****P<0.0001 (*n*=4–6/group). See Supplementary Figure S6

Mitochondrial dysfunction often leads to increased oxidative stress and contributes to ageing. As our data showed a higher abundance of OXPHOS complex proteins in aged *Casp2*^*−/−*^ *versus* WT mice, we investigated whether there was any difference in mitochondrial size, number and function *in vivo.* Electron microscopy revealed a small but significant decrease in mitochondrial density and size in hepatocytes from young *Casp2*^*−/−*^ *versus* WT mice (Figures 5a and b). A significant decrease in mtDNA copy number was observed with age (Figure 5c), but this did not correlate with the mitochondrial number. The abundance of the five OXPHOS complexes resolved by BN-PAGE and detected by immunoblotting was not affected (Supplementary Figure S6A), suggesting that proteomic differences may result in altered OXPHOS polypeptide stoichiometry in aged *Casp2*^*−/−*^ mice. Measurements of the enzyme function of all five complexes and citrate synthase revealed significantly decreased activity of both citrate synthase and complex III in aged and young *Casp2*^*−/−*^ relative to the WT young mice (Figure 5d). This suggests that complex III activity is specifically affected by loss of Casp2 activity. In contrast, a significant increase in citrate synthase activity was observed with age in *Casp2*^*−/−*^ mice (Figure 5d), which may be a compensatory response to the decreased activity of complex III. In aged mice, activities of complexes II and V increased in both genotypes, and the activity of complex II was significantly higher in the aged *Casp2*^*−/−*^ *versus* WT mice (Figure 5d). Previously, we found no difference in mitochondrial function upon loss of Casp2 in primary MEFs,^11^ which was confirmed here by measurement of the respiratory complex activities (Supplementary Figures S6B and C). However, *Casp2*^*−/−*^ MEFs trended (*P*=0.08) towards increased spare respiratory capacity.

### Loss of Casp2 alters glucose homeostasis in aged mice

**Figure 6:**
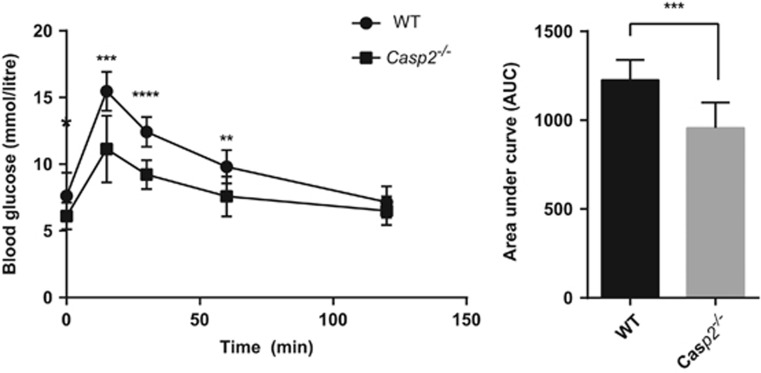
*Casp2*^*−/−*^ mice show resistance to age-induced glucose intolerance. IPGTT (left) and AUC (right) for aged WT and *Casp2*^*−/−*^ mice. Values are means±S.D. *P<0.05; **P<0.01; ***P<0.001, ****P<0.0001 (*n=*9–10)

To investigate whether glucose homeostasis was altered by loss of Casp2 as indicated from the serum metabolomics, aged mice were subjected to intraperitoneal glucose tolerance testing (IPGTT). Blood glucose was measured in fasted (6 h) aged WT and *Casp2*^*−/−*^ mice at baseline and 15, 30, 60 and 120 min after intraperitoneal injection of glucose. Interestingly, fasting blood glucose levels (6.1±1.01 mmol/l) and IPGTT were significantly reduced in aged *Casp2*^*−/−*^ mice (Figure 6). Increased fasting blood glucose and impaired glucose tolerance are normally associated with ageing, thus these results suggest that *Casp2*^*−/−*^ mice show resistance to age-induced glucose intolerance.

## Discussion

Using comparative profiling, we investigated reprogramming of metabolism and homeostasis during normal animal ageing and the role of Casp2 in these processes. Ageing was associated with upregulation of enzymes involved in carbohydrate and amino-acid metabolism, downregulation of numerous mitochondrial and ribosomal proteins and altered mitochondrial function. A number of age-related changes were deregulated in *Casp2*^*−/−*^ mice, suggesting the importance of Casp2 in maintaining metabolic and energy homeostasis with age. Furthermore, young *Casp2*^*−/−*^ mice had levels of some proteins and metabolites already characteristic of those found in aged WT mice, including low NADPH and altered mitochondria function. This suggests early onset of energy impairment in *Casp2*^*−/−*^ mice that likely contributes to increased oxidative stress,^11^ reduced stress tolerance and early onset ageing. Intriguingly, aged *Casp2*^*−/−*^ mice had reduced fasting blood glucose and improved glucose tolerance, suggesting that Casp2 is important for glucose homeostasis.

Some age-related changes to the proteome and metabolome occur in an attempt to improve longevity, perhaps by conserving energy to assist with repair of molecular and cellular damage. Impaired protein homeostasis (proteostasis) is characteristic of ageing. Our finding of decreased ribosomal proteins during ageing is indicative of altered proteostasis that might reduce protein synthesis as was recently found in *Caenorhabditis elegans*.^32^ Reduced protein translation is associated with increased longevity and is likely a beneficial change that occurs with ageing.^32^ The substantial reduction in the number of ribosomal proteins that are downregulated during ageing in *Casp2*^*−/−*^ mice suggests that Casp2 has a role in regulating proteostasis. Protein synthesis is an energy-consuming process and failure to decrease protein translation would likely result in increased stress in the *Casp2*^*−/−*^ mice, possibly contributing to the early-onset ageing.

Impaired glucose tolerance and increased fasting blood glucose levels are associated with ageing. Our study shows a decline in serum glucose with age in non-fasted mice, consistent with another study.^26^ Serum glucose was further reduced in both young and aged *Casp2*^*−/−*^ *versus* WT mice. Interestingly, fasting blood glucose was lower in aged *Casp2*^*−/−*^ mice, and glucose tolerance was improved. This may be attributable to the previous observation of reduced FoxO1 and FoxO3 transcription factors in aged *Casp2*^*−/−*^ mice.^11^ FoxO transcription factors are important mediators of insulin signalling and have been shown to promote hepatic glucose production.^33^ Furthermore, liver-specific FoxO1 and triple FoxO1/3/4 knockouts have improved glucose tolerance and reduced fasting and non-fasting blood glucose.^34, 35^ Altered mitochondria function has also been linked with reduced hepatocyte glucose production.^36^ Increased levels of ROS in glutathione peroxidase 1 knockout mouse have been shown to enhance insulin sensitivity and improve glucose tolerance.^37^ Thus increased ROS in aged *Casp2*^*−/−*^ mice may also contribute to improved glucose tolerance.

Our study has identified 4-hydroxyproline as a potential biomarker of ageing, being the metabolite with greatest change in abundance in both the liver and serum. Declines in 4-hydroxyproline may be associated with increased collagen degradation during ageing and/or decreases in proline.^38^ Decreases in amino acids serine, alanine and methionine in serum are consistent with mouse and human studies^27, 39, 40^ and may also be potential biomarkers of ageing. Increases in plasma FFAs, similar to our findings, have been previously implicated as biomarkers of ageing.^27, 41^ Specifically, decreases in serine and increases in essential fatty acid eicosapentoate (EPA; 20:5n3) have been identified in the human study of 6055 individuals to be part of a panel of 22 independent metabolites associated with age.^39^

Decreased citrate synthase and complex III activity in young *Casp2*^*−/−*^ mice suggests impaired mitochondrial function that may lead to increased ROS contributing to premature ageing. This finding is supported by a recent study using hepatocytes isolated from 12-month-old mice which suggests that loss of Casp2 results in accelerated age-dependent changes to mitochondrial ROS production, potentially through altered complex III activity.^42^ Decreased activities of complexes III and IV have been previously observed with ageing^43, 44, 45^ as has decreased abundance of complex III protein subunits.^23^ Increased mitochondrial size, citrate synthase activity and higher complex II activity in aged *Casp2*^*−/−*^ mice are likely to be a compensatory response to subtle energy changes in young mice.

Decreased FFAs are likely the result of decreased ACC1 protein and may be a direct reflection of triglyceride metabolism. In addition, it is likely that low NADPH levels in *Casp2*^*−/−*^ mice has a major role in the impaired antioxidant response and decreased liver FFAs. As NADPH has previously been reported to negatively regulate Casp2 activity,^14^ we hypothesized that a feedback loop could contribute to the reduced levels of NADPH in young *Casp2*^*−/−*^ mice but found no evidence to support this. Alternatively, altered NADPH levels may be a direct result of altered mitochondrial function.

G3P is a central metabolite of glucose and lipid metabolism, and OXPHOS is likely interconnected with low NADPH, FFA and liver triglycerides. As G3P is an intermediate of glycolysis, decreased G3P may reflect a decrease in glycolytic activity. Low levels of G3P in our study are likely the result of decreased levels of DAK enzyme that is involved in generating the G3P precursor DHAP.^46^ Alternatively, G3P can be generated from glycerol following its release from triglycerides during lipolysis; however, no differences in glycerol levels were detected in our metabolomics screen. Decreased G3P may also result in lower OXPHOS activity. Conversion of G3P to DHAP by mitochondrial G3PDH in the mitochondrial inner membrane, results in recycling of FADH_2_ to FAD that is necessary for driving the flow of electrons via Coenzyme Q to Coenzyme QH_2_ for utilization by OXPHOS.^47^ G3P is also a precursor for serine biosynthesis, and thus the low levels of serine, and subsequently glycine, may be a consequence of decreased G3P. Alternatively, serine is also generated from glucose, and decreased levels may reflect a decrease in glycolytic rate. Importantly, serine is also a gluconeogenic metabolite, being converted to pyruvate by SDS. Also a precursor of glycine biosynthesis, the essential amino-acid threonine was one of the liver metabolites most significantly reduced in young *Casp2*^*−/−*^ *versus* WT and was decreased with WT ageing. Expression levels of SDS protein have been shown to strongly correlate with its level of enzymatic activity.^30^ We hypothesized that increases in SDS protein (and assumed activity) may result in increased degradation of serine and threonine resulting in decreased abundance of these metabolites.

In conclusion, our data provide evidence that Casp2 regulates age-dependent homeostatic changes and stress response pathways, including NADPH levels, OXPHOS and ribosomal function. Intriguingly, Casp2 also modulates amino acid, fatty acid and glucose metabolism and influences glucose homeostasis. Consistent with altered mitochondrial function, Casp2 appears to regulate the activity of complex III, which may contribute to increased ROS production and oxidative stress observed in *Casp2*^*−/−*^ mice. This study provides a global overview as to how the loss of Casp2 contributes to the major physiological processes that contribute to altered metabolism, mitochondria function and the early progression of ageing.

## Materials and Methods

### Animals, cell culture and sample collection

Male *Casp2*^*−/−*^ mice on a C57BL/6J background^11^ were used for experimental studies at 6–9 weeks and 18–24 months of age. Primary MEFs were derived from embryos at embryonic day 13.5 as previously described.^11^ Ethics for approval for research using animals was obtained from SA Pathology/Central northern Adelaide health Services Animal Ethics Committee, in accordance with National Health and Medical Research Council of Australia guidelines. Mice were housed in pathogen-free conditions with a 12-h light:dark cycle and fed *ad libitum* on standard chow. Harvested tissues and serum were snap-frozen in liquid nitrogen (N_2_) and kept at −80 °C until analysed. See Supplementary Experimental Procedures for full methods.

### Liver proteomic analysis

Proteins isolated from perfused liver tissue from young and aged WT and *Casp2*^*−/−*^ mice were analysed using 4-plex iTRAQ labelling by the Australian Proteome Analysis Facility (APAF, Sydney, NSW, Australia). See Supplementary Experimental Procedures for full methods.

### Liver and serum metabolomics analysis

Metabolites of the liver and serum from young and aged WT and *Casp2*^*−/−*^ mice were extracted and analysed by Metabolomics Australia (Melbourne, VIC, Australia). See Supplementary Experimental Procedures for full methods.

### Enrichment analysis of biological processes and pathways

See Supplementary Experimental Procedures for full methods.

### Mitochondria content, size and DNA copy number

Liver tissue samples were examined by transmission electron microscopy to determine the density and size of mitochondria in hepatocytes and were analysed by qPCR to determine the mitochondrial DNA copy number genes. See Supplementary Experimental Procedures for full methods.

### Mitochondrial OXPHOS complex and enzyme assays

Enzyme assays for citrate synthase, complexes I, II, III, IV and V were carried out on isolated mitochondria in 1-ml cuvette at 30 °C using a Perkin Elmer lambda 35 dual beam spectrophotometer (Waltham, MA, USA). See Supplementary Experimental Procedures for full methods.

### Cellular bioenergetics by Seahorse bioanalysis

The cellular bioenergetics profiling of WT and *Casp2*^*−/−*^ MEFs was assessed using the Seahorse X24 Flux Analyzer (Seahorse Bioscience, North Billerica, MA, USA). See Supplementary Experimental Procedures for full methods.

### Liver and serum biochemistry

See Supplementary Experimental Procedures for full methods.

### Immunoblotting

See Supplementary Experimental Procedures for full methods.

### Intraperitoneal glucose tolerance test

Glucose tolerance tests were performed on food-deprived (6 h) non-anesthetized mice. Glucose measures were obtained from whole-tail vein blood using an automated glucometer at baseline and at 15, 30, 60 and 120 min after intraperitoneal injection of 1 mg/kg glucose.

### Statistical analysis and data visualization

Statistical analysis was performed using the GraphPad Prism software (v 6.0, GraphPad Software Inc., La Jolla, CA, USA) or within Microsoft Excel (Microsoft, Redmond, WA, USA). Data are expressed as means±S.D. or means±S.E.M. For pair-wise comparisons of metabolomics data, a two-tailed unpaired *t-*test with Welch's correction was used. Unless indicated otherwise, statistical analysis performed by the Students *t*-test or *t-*test with Welch's correction. Heat maps and Venn diagrams were generated using the online software. See Supplementary Experimental Procedures for full methods.

## Abbreviations

ACC1: acetyl-CoA carboxylase 1
Casp2: caspase-2
CYP: cytochrome P450
DAK: biofunctional ATP-dependent dihydroxyacetone kinase/FAD-AMP lyase (cyclizing)
FFA: free fatty acid
G3P: glycerol-3 phosphate
GYS: glycogen synthase
IPGTT: intraperitoneal glucose tolerance test
NAD/NADH: nicotinamde-adenine dinucleotide
NADP/NADPH: nicotinamide-adenine dinucleotide phosphate
OXPHOS: oxidative phosphorylation
PPP: pentose-phosphate pathway
ROS: reactive oxygen species
SDS: L-serine dehydratase/L-threonine deaminase
