# Supplementary material for: Age-related proteostasis and metabolic alterations in Caspase-2-deficient mice
Source: Cell Death Dis. 2015 Jan 22;6(1):e1597–. doi: 10.1038/cddis.2014.567 (PMC4669765; doi:10.1038/cddis.2014.567)
Supplement: Supplementary Information [file cddis2014567x1.pdf]

SUPPLEMENTAL TABLE LEGENDS

Table S1 related to Figure 1: Common proteins between groups of interest identified by Four-way Venn diagram analysis of high confidence (probability >95%, minimum 2 unique peptides) liver proteins detected by proteomics analysis of young (6-9 week) and aged (18-24 month) WT and *Casp2*$^{-/-}$ mice. Cellular component, biological process and molecular function are generated in ProteinPilot v4.2b (AB Sciex).

Table S2 related to Figure 1: Differentially abundant liver proteins identified by comparative proteomics. Liver proteins identified as being differentially expressed $p< 0.05$ or those with a ratio ≥1.2 or ≤ 0.83 and $p<0.1$ during ageing of WT or *Casp2*$^{-/-}$, and between genotypes in young (6-8 week) and aged (18-24 month) old . Green cells highlight protein ratios ≤ 0.83 or that are decreased with *p-value* <0.05; Red cells highlight protein ratios ≥1.2 or that are increased with $p< .05$; Yellow cells highlight $p< 0.05$; Dark-orange cells highlight $p <0.1$. Statistical significance determined by ANOVA.

Table S3 related to Figure2: Global metabolomics of liver and serum during ageing of WT and *Casp2*$^{-/-}$ mice. Contains details of total (known and unknown) metabolites identified (individual measured (normalized) values) and differentially abundant metabolites (fold-change values). For differential metabolites, Red and green shading highlight metabolites identified as being differentially increased or decreased in abundance respectively. Where available CAS number, KEGG number and human metabolome database (HMDB) ID (version 3.5) provided.

Table S4 related to Figure 3: Details of individual proteins and the enriched biological processes (PANTHER) from liver proteins differentially abundant during ageing of WT and *Casp2*$^{-/-}$ mice and between genotype of each age group. Enrichment analysis performed using using DAVID Bioinformatics resources 6.7 (http://david.abcc.ncifcrf.gov).

Table S5 related to Figure 3: Details of individual proteins and the enriched biochemical pathways (KEGG) from liver proteins differentially abundant during ageing of WT and *Casp2*$^{-/-}$ mice and between genotype of each age group. Enrichment analysis performed using using DAVID Bioinformatics resources 6.7 (http://david.abcc.ncifcrf.gov).

SUPPLEMENTAL FIGURE LEGENDS

Figure S1 related to Figure 1: Differential abundance of liver proteins in young *Casp2*$^{-/-}$ versus young WT mice and aged *Casp2*$^{-/-}$ versus aged WT mice. (A) Heatmap columns represent abundance of proteins in individual young *Casp2*$^{-/-}$ mice versus averaged young WT mice. (B) Left hand side shows heat map of liver proteins differentially altered between individual aged *Casp2*$^{-/-}$ mice versus aged WT mice. Right hand side shows heat map of the averaged liver proteins differentially altered during ageing of WT and *Casp2*$^{-/-}$ mice (n = 4/group). Left hand side heat map columns represent abundance of proteins in individual aged *Casp2*$^{-/-}$ mice versus averaged aged WT mice (n=4/group). Green box highlights example referred to in text. Right hand side heat map columns represent average abundance of proteins altered during ageing of WT and *Casp2*$^{-/-}$ mice (n=4/group).For a complete list of differentially abundant proteins refer to Table S2.

Figure S2 related to Figure 2: Differential abundance of metabolites in liver and serum during ageing of WT and *Casp2*$^{-/-}$ mice. Heat maps of (A-D) liver and (E-H) serum metabolites differentially abundant in (A,E) young *Casp2*$^{-/-}$ versus young WT, (B,F) aged *Casp2*$^{-/-}$ versus aged WT and during ageing of (C,G) WT and (D,H) *Casp2*$^{-/-}$ mice. Heatmaps display the number of increased (red) or decreased (blue) metabolites. Heatmap columns represent abundance of metabolites in individual mice relative to average of comparison group. Individual values can be found in Table S3.

Figure S3 related to Figure 3: Enrichment of biological processes and biochemical pathways in liver proteome during ageing. (A-D) Biological processes (PANTHER) enriched in differentially abundant liver protein during ageing of (A) WT and (B) *Casp2*$^{-/-}$ mice and between (C) young and (D) aged WT and *Casp2*$^{-/-}$ mice. (E,F) Biochemical pathways enriched in liver during ageing of (E) WT and (F) *Casp2*$^{-/-}$ mice. Dotted line represents cut-off for significance of [–log10(*p*-value)] with Benjamini correction. Enrichment of Protein Analysis Through Evolutionaroy Relatinoships (PANTHER) biological process and Kyoto Encyclopedia of Genes and Genomes (KEGG) pathway was performed using DAVID Bioinformatics resources 6.7 (http://david.abcc.ncifcrf.gov). Details of individual proteins included in each enriched biological process or biochemical pathway can be found in Table S4 and S5.

Figure S4: Pathway analysis global liver metabolomics during ageing of WT and *Casp2*$^{-/-}$ mice. Summary plots for metabolite set enrichment analysis of global metabolomics differences in liver during ageing of WT (A) and *Casp2*$^{-/-}$ (B) mice and differences between

young *Casp2*$^{-/-}$ and WT mice (C). Plots are ranked according to *p*-value. Analysis performed using MetaboAnalyst 2.0 (http://www.metaboanalyst.ca/MetaboAnalyst).

Figure S5: Pathway analysis of global serum metabolomics during ageing of WT and *Casp2*$^{-/-}$ mice. Summary plots for metabolite set enrichment analysis of global metabolomics differences in serum during ageing of WT (A) and *Casp2*$^{-/-}$ (B) mice and differences between young *Casp2*$^{-/-}$ and WT mice (C). Plots are ranked according to *p*-value.). Plots are ranked according to *p*-value. Analysis performed using MetaboAnalyst 2.0 (http://www.metaboanalyst.ca/MetaboAnalyst/).

Figure S6 related to Figure 5: Mitochondria function, turnover OXPHOS complex abundance in MEFs and liver

(A) Nu-BLUE page analysis of liver mitochondrial proteins for total abundance of respiratory chain complexes of aged WT and *Casp2*$^{-/-}$ liver mitochondria. (B,C) Seahorse Bioanalyzer analysis of primary WT and *Casp2*$^{-/-}$ mice to determine (B) basal bioenergetics and (C) mitochondria function *in vitro.* Values are means ± SEM. ** p<0.001.

SUPPLEMENTAL EXPERIMENTAL PROCEDURES

Animals, cell culture and sample collection

Male *Casp2*$^{-/-}$ mice on a C57BL/6J background [1] were used for experimental studies at 6-9 weeks and 18-24 months of age. Primary MEFs were derived from embryos at embryonic day 13.5 as previously described [1]. Ethics for approval for research using animals was

obtained from SA Pathology/Central northern Adelaide health Services Animal Ethics Committee, in accordance with National Health and Medical Research Council of Australia guidelines. Mice were housed in pathogen-free conditions with a 12 hour light:dark cycle and fed *ad libitum* on standard chow. Harvested tissues and serum were snap-frozen in liquid nitrogen (N_2) and kept at -80 ^{o}C until analysed. For proteomics, livers were rapidly perfused with PBS prior to harvesting. Mouse liver mitochondria were isolated from freshly harvested liver tissue using a standard procedure by homogenisation followed by differential centrifugation [2]. The mitochondrial protein concentration was measured using the BCA assay [3].

Liver proteomic analysis

Perfused liver tissue (50 mg) was homogenized in ice-cold homogenization buffer (50 mM Hepes pH 7.1, 0.1% (w/v) SDS, 200 mM NaCl, 1 mM EDTA) containing 1x Halt™ Protease and Phosphatase Inhibitor Cocktail (Thermo Scientific) then incubated with agitation at 4 ^{o}C for 30 min. Samples taken through 3 rounds of LN_2 freeze thawing then sonicated for 3 x 10 sec each. Homogenates were clarified by centrifugation at 1,500 g^{-1} for 10 min, 4 ^{o}C. Supernatants collected and further clarified by centrifugation at 16 000 g^{-1} for 30 min, 4 ^{o}C. Protein concentration determined by Bradford (BioRad). Samples stored -70 ^{o}C and transported by overnight dry-ice shipment for processing and analysis by the Australian Proteome Analysis facility (APAF, Sydney, NSW, Australia). Four individual livers were processed for each group. Samples were divided into six 4-plex iTRAQ experiments and an internal standard prepared by combining equal amounts of protein from 4 young WT mice to allow for analysis of data across all six experiments. Samples (100 µg) were reduced with TCEP, alkylated with MMTS, digested with trypsin then peptides labelled according to

standard operating procedures (APAF, Australia). Labelled peptides were cleaned and fractionated by SCX HPLC as previously described [4].

LC-MS/MS and data analysis

SCX fractions resuspended in 0.1% TFA and 2% acetonitrile were loaded onto a reverse phase peptide Captrap (Michrom Bioresources) and desalted at 10 µl per minute for 13 minutes then trap switch on-line with a C_{18} reverse phase columns (150 µM x 10 cm, 3 µm, 200A SGE ProteCol C_{18} column) for NanoLC separation (Eksigent Tempo nanoLC system buffer A (0.1% formic acid). Peptides were eluted with a three-step linear gradient of 5-90% buffer B (90% ACN and 0.1% formic acid) over 120 min at 500 nL/min. This was followed by 15 min of 100% buffer B, washout and re-equilibration with buffer A for 30 min before next sample injection. Peptides were then electrosprayed and analyzed on a Qstar Elite MS/MS (AB Sciex) using positive ion nanoflow electrospray analysis in an information dependant acquisition mode (IDA). In IDA mode a TOFMS survey scan was acquired (m/z 370-1600, 0.5 second), with the three most intense multiple charged ions (counts > 70) in the survey scan sequentially subjected to MS/MS analysis. MS/MS spectra were acquired in the mass range m/z 100-1600. MS/MS data were analysed against the UniProtKB/SwissProt *Mus musculus* database (release 2012_01, 16 473 entries) using ProteinPilot v4.2b (AB Sciex) software. The Paragon search algorithm was used in Thorough ID search effort for peak-picking and database matching. Data were searched considering cysteine alkylation (MMTS), trypsin digestion and biological modifications selected in ID Focus. The detected protein threshold (unused ProtScore) was set as larger than 1.3 (better than 95% confidence). Data were normalized by auto bias-correction and ratios natural logarithm transformed for further statistical analysis. Only high confidence proteins (p > 95%) identified by 2 or more unique

peptides were selected for further analysis. Statistical comparisons between groups were performed by ANOVA.

Liver and serum metabolomics analysis

Metabolomics analysis of liver and serum of young and aged WT and *Casp2*$^{-/-}$ mice was performed by Metabolomics Australia (Melbourne, Vic, Australia). Frozen samples were sent by overnight dry ice shipment for processing and analysis. Liver and serum metabolites were analysed by LC-MS amine quantitation, global (untargeted) GC-MS analysis of polar metabolites and targeted GC-MS analysis of fatty acids. In brief, liver samples were extracted using a mixture of chloroform:methanol:water (1:3:1) and a quantitative internal standard ($^{13}C_6$-sorbitol/$^{13}C_{15}N$-Valine in water, 0.2 mg mL^{-1}). Samples were split into aliquots for each GC-MS and LC-MS analysis. For GC-MS, samples were dried *in vacuo* prior to derivatisation with *N,O-bis*-(trimethylsilyl) trifluoroacetamide (BSTFA) for untargeted polar-metabolite analysis or were reconstituted in 2:1 chloroform:methanol then derived with Meth-Prep™ II (Grace Davison Discovery) for targeted fatty-acid analysis. GC-MS was performed using a Gerstel 2.5.2 autosampler, a 7890A Agilent GC and a 5975C Agilent quadrupole MS (Agilent, Santa, Clara, USA) with a 30 m VF-5MS column with 0.2 µm film thickness and a 10 m Integra guard column (Varian, Inc, Victoria, Australia). Both chromatograms and mass spectra were evaluated using either the Analyzer Pro Deoconvolution Program (Spectralworks, UK) or Agilent MassHunter Workstation Software, Quantitative Analysis, Version B.05.00/Build 5.0.291.0 for GC-MS. For polar metabolites, mass spectra of eluting compounds were identified using the public domain mass spectra library of Max-Plank-Institute for Plant Physiology, Golm, Germany (http://csbdb.mpimp-golm.mpg.de/csbdb/dbma/msri.html) and the *in-house* Metabolomics Australia mass spectral

library. For fatty acids, the retention times and mass spectra were identified quantified as previously described [5]. Amino acid derivatisation and LC-MS amine quantitation was performed according to published methods [6]. LC-MS was performed on an Agilent 1200 LC-system coupled to an Agilent 6410 ESI-Triple Quadrupole-MS (Agilent, Santa, Clara, USA) with an Agilent Zorbax Eclipse XDB-C18 Rapid Resolution HT 2.1 x 50 mm, 1.8 µm column.

Enrichment analysis of biological processes and pathways

Enrichment analysis of proteins identified as being significantly altered in abundance was performed using the web-based software DAVID (Database for Annotation, Visualization and Integrated Discovery) Bioinformatics Resources 6.7 (http://davd.abcc.ncifcrf.gov/)[7]. Enriched biological processes were identified from the PANTHER database and enriched pathways from the KEGG (Kyoto Encyclopedia of Genes and Genomes) by DAVID using the default EASE scoring system and Benjamini correction of *p*-values with a *p*-value <= 0.05 considered to be strongly enriched.

Metabolite set enrichment analysis was performed against the 'Metabolite pathway associated metabolite set' library that contains 88 metabolite sets based on normal metabolic pathways using the web-based tool, MetaboAnalyst 2.0 (http://www.metaboanalyst.ca/MetaboAnalyst/)[8].

Mitochondria content, size and DNA copy number

Liver tissue samples for transmission electron microscopy (TEM) examination were immediately fixed following isolation in 4% (v/v) paraformaldehyde in 0.1 M phosphate

buffer (pH 7.4) to which 2% (v/v) glutaraldehyde. Tissue processing, sectioning and staining was performed as described [9]. Sections were viewed with a PHILIPS CM100 TEM equiped with a SIS MegaviewII CCD Camera and images captured using the analysis software. Digital images of 3-5 fields of view per sample were analysed using Image J software (NIH; http: imagej.nih.gov/ij/) for measurement of mitochondria density and size in hepatocytes. Mitochondrial density was measured as the number of mitochondria per total area (μm^2) of hepatocyte cytoplasm. Mitochondrial size was computed as the total area (μm^2).

Mitochondrial DNA copy number genes were measured on DNA isolated from mouse liver mitochondria using the DNeasy Blood & Tissue Kit (Qiagen). MtDNA was used as a template in a qPCR that was performed using mouse specific primers on a Corbett Rotorgene 3000 using Platinum UDG SYBR Green mastermix (Invitrogen).

Mitochondrial oxidative phosphorylation (OXPHOS) complex and enzyme assays

Enzyme assays were carried out in a 1 ml cuvette at 30 °C using a Perkin Elmer lambda 35 dual beam spectrophotometer as described previously [10]. For mitochondrial oxidative phosphorylation complex assays, mitochondria in STE with protease inhibitors were disrupted by two to four cycles of freezing in dry ice/ethanol and thawing at 30 °C. Citrate synthase was assayed by standard procedures [11]. Complex I was assayed as the rotenone-sensitive rate of NADH reduction of CoQ_1, Complex II by the thenoyltrifluoroacetone-sensitive rate of succinate reduction of 2,6-dichlorophenolindophenol. Combined Complex II and Complex III activity was assayed as the myxothiazol-sensitive rate of reduction of ferricytochrome c by succinate. Complex III was measured as the myxothiazol-sensitive rate of reduction of ferricytochrome c by CoQ_2 and Complex IV was measured as the cyanide-sensitive oxidation of ferrocytochrome c. Complex V was assayed as the oligomycin-

sensitive rate of ATP hydrolysis, measured by coupling ADP production to NADH oxidation by a linked enzyme assay. Briefly, ATP hydrolyzed to ADP by Complex V was reconverted to ATP through reaction with phosphoenolpyruvate catalyzed by pyruvate kinase, with the pyruvate produced coupled to NADH oxidation by lactate dehydrogenase

Cellular bioenergetics by Seahorse Bioanalysis

The cellular bioenergetics profiling of WT and *Casp2*$^{-/-}$ MEFs was assessed using the Seahorse X24 Flux Analyzer (Seahorse Bioscience). Cells were seeded into a XF24 microplate at a density of 25,000 cells per well and analysed the following day. To evaluate the basal bioenergetics and mitochondrial function, cells were washed and incubated in 600µl unbuffered DMEM (containing 25mM glucose, 1mM pyruvate and 1mM glutamate) pH 7.4, at 37°C in a non-CO_2 incubator (1h prior to bioenergetics assessment). Three basal oxygen consumption rate (OCR) measurements were performed using the Seahorse analyzer, and measurements were repeated following injection of oligomycin (1µM), FCCP (1µM) and Antimycin A (1µM). Basal extracellular acidification rate (ECAR) was determined from data collected at basal measurement points. Calculations of respiratory parameters of mitochondrial function were performed as previously described (McGee et al., 2011). Following completion of the assay, cell number was determined using the CyQuant® Cell Proliferation Assay kit (Molecular Probes) according to manufacturer's instructions.

Liver and serum biochemistry

Triglycerides and liver marker enzymes alanine transaminase (ALT), aspartate aminotransferase (AST) and lactate dehydrgonase (LDH) were determined in serum by

automated analysis (SA Pathology). Free-fatty acids (liver and serum), triglycerides, NADP, NADPH, NAD, NADH and activity of G6PDH and G3PDH were determined in liver using commercially available assay kits (BioVision; Cayman Chemical).

Immunoblotting

Total liver proteins (50 µg) were resolved by SDS-PAGE and transferred to PVDF membrane. Mitochondria proteins (75 µg) were resolved by BN-PAGE as described previously [12]. Specific proteins were detected using mouse monoclonal antibodies: NDUFA9, Complex II subunit 70 kDa, Complex III subunit core 2 (UQCRC2), COXI, COXIV and porin (MitoSciences), MnSOD (BD Biosciences), ND1 (Santa Cruz Biotechnology); mouse polyclonal antibodies: SDS (#ab68536, Abcam); rabbit polyclonal antibodies: DAK (#ab137623, Abcam), ACC1 and GYS (#4190 and #3893, Cell Signaling Technology).

Intraperitoneal glucose tolerance test (IPGTT)

Glucose tolerance tests were performed on food-deprived (6 h) non-anesthetized mice. Glucose measures were obtained from whole-tail vein blood using an automated glucometer at baseline and 15, 30, 60 and 120 min after intraperitoneal injection of 1 mg/kg glucose.

Statistical analysis and data visualisation

Statistical analysis was performed using GraphPad Prism software (v 6.0) or within Microsoft Excel. Data are expressed as means ± SD or means ± SEM. For pair-wise comparisons of

metabolomics data a two-tailed unpaired *t*-test with Welch's correction was used. Unless indicated otherwise, statistical analysis performed by Students *t*-test or *t*-test with Welch's correction.

For the generation of heatmaps, online software CARMAWeb 1.5 [13] was used. Heatmaps were generated from natural log transformed proteomics data and $\log_2$ transformed metabolomics data. Venn diagrams were generated using online software Venny [14].

SUPPLEMENTAL REFERENCE

1. Shalini S, Dorstyn L, Wilson C, Puccini J, Ho L, Kumar S. Impaired antioxidant defence and accumulation of oxidative stress in caspase-2-deficient mice. *Cell Death Differ* 2012, **19**(8)**:** 1370-1380.

2. Chappell JB, Hansford RG. *Preparation of mitochondria from animal tissues and yeast*. Butterworths: London, 1972.

3. Smith PK, Krohn RI, Hermanson GT, Mallia AK, Gartner FH, Provenzano MD*, et al.* Measurement of protein using bicinchoninic acid. *Anal Biochem* 1985, **150**(1)**:** 76-85.

4. Yang J, MacDougall ML, McDowell MT, Xi L, Wei R, Zavadoski WJ*, et al.* Polyomic profiling reveals significant hepatic metabolic alterations in glucagon-receptor (GCGR) knockout mice: implications on anti-glucagon therapies for diabetes. *BMC Genomics* 2011, **12:** 281.

5. Olmstead IL, Hill DR, Dias DA, Jayasinghe NS, Callahan DL, Kentish SE*, et al.* A quantitative analysis of microalgal lipids for optimization of biodiesel and omega-3 production. *Biotechnol Bioeng* 2013, **110**(8)**:** 2096-2104.

6. Boughton BA, Callahan DL, Silva C, Bowne J, Nahid A, Rupasinghe T*, et al.* Comprehensive profiling and quantitation of amine group containing metabolites. *Anal Chem* 2011, **83**(19)**:** 7523-7530.

7. Huang da W, Sherman BT, Lempicki RA. Systematic and integrative analysis of large gene lists using DAVID bioinformatics resources. *Nat Protoc* 2009, **4**(1)**:** 44-57.

8. Xia J, Mandal R, Sinelnikov IV, Broadhurst D, Wishart DS. MetaboAnalyst 2.0--a comprehensive server for metabolomic data analysis. *Nucleic Acis Res* 2012, **40**(Web Server issue)**:** W127-133.

9. Steenks M, van Baal MC, Nieuwenhuijs VB, de Bruijn MT, Schiesser M, Teo MH*, et al.* Intermittent ischaemia maintains function after ischaemia reperfusion in steatotic livers. *HPB (Oxford)* 2010, **12**(4)**:** 250-261.

10. Davies SM, Poljak A, Duncan MW, Smythe GA, Murphy MP. Measurements of protein carbonyls, ortho- and meta-tyrosine and oxidative phosphorylation complex activity in mitochondria from young and old rats. *Free Radic Biol Med* 2001, **31**(2)**:** 181-190.

11. Shepherd D, Garland PB. The kinetic properties of citrate synthase from rat liver mitochondria. *Biochem J* 1969, **114**(3)**:** 597-610.

12. Wittig I, Braun HP, Schagger H. Blue native PAGE. *Nat Protoc* 2006, **1**(1)**:** 418-428.

13. Rainer J, Sanchez-Cabo F, Stocker G, Sturn A, Trajanoski Z. CARMAweb: comprehensive R- and bioconductor-based web service for microarray data analysis. *Nucleic Acids Res* 2006, **34**(Web Server issue)**:** W498-503.

14. Oliveros JC. VENNY. An interactive tool for comparing lists with Venn Diagrams. 2007 [cited]Available from: http://bioinfogp.cnb.csic.es/tools/venny/index.html

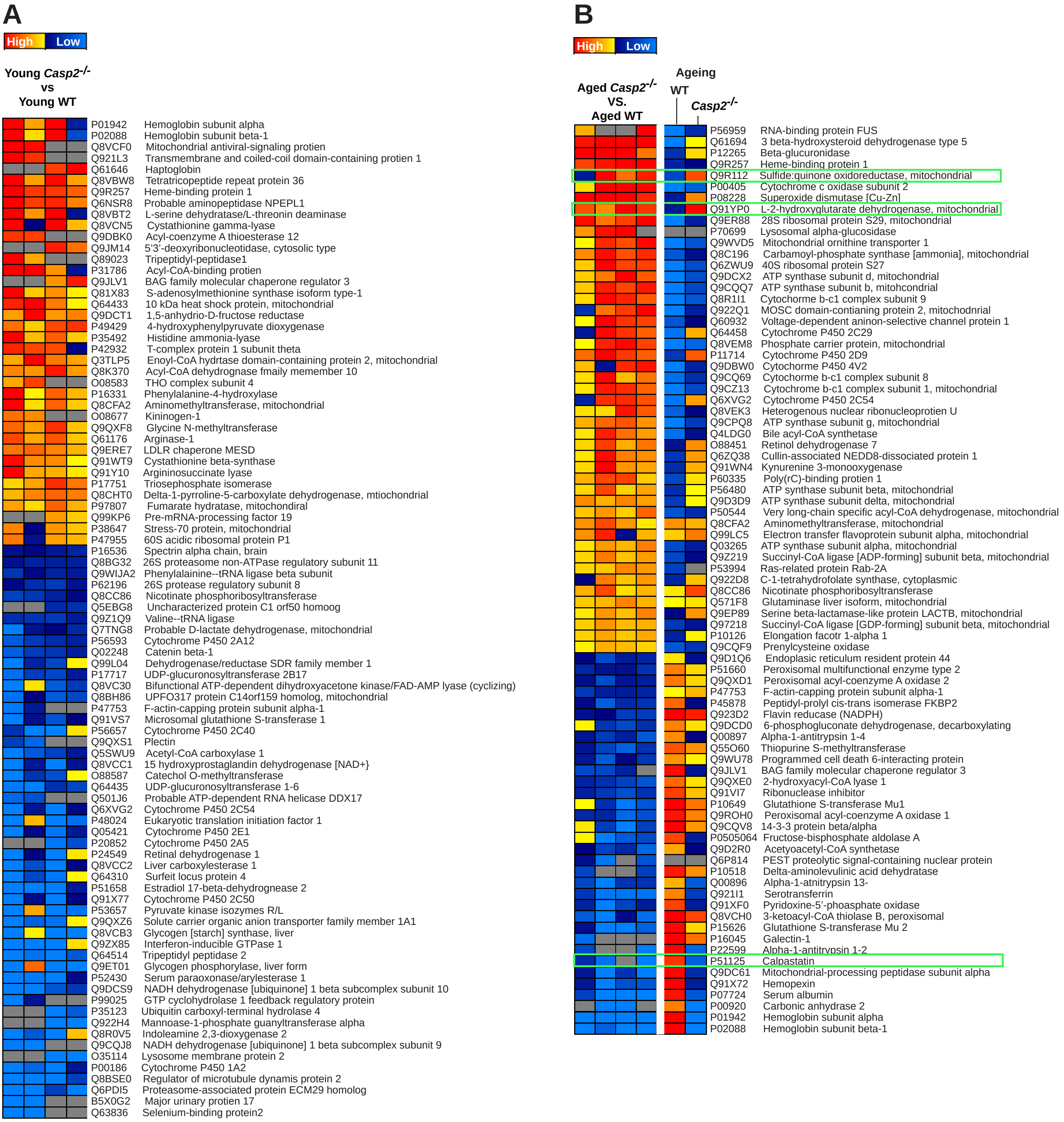
A
High
Low
Young Casp2-/- vs Young WT
P01942 Hemoglobin subunit alpha
P02088 Hemoglobin subunit beta-1
Q8VCF0 Mitochondrial antiviral-signaling protien
Q921L3 Transmembrane and coiled-coil domain-containing protein 1
Q61646 Haptoglobin
Q8VBW8 Tetratricopeptide repeat protein 36
Q9R257 Heme-binding protein 1
Q6NSR8 Probable aminopeptidase NPEPL1
Q8VBT2 L-serine dehydratase/L-threonin deaminase
Q8VCN5 Cystathionine gamma-lyase
Q9DBK0 Acyl-coenzyme A thioesterase 12
Q9JM14 5'3'-deoxyribonucleotidase, cytosolic type
Q89023 Tripeptidyl-peptidase1
P31786 Acyl-CoA-binding protien
Q9JLV1 BAG family molecular chaperone regulator 3
Q81X83 S-adenosylmethionine synthase isoform type-1
Q64433 10 kDa heat shock protein, mitochondrial
Q9DCT1 1,5-anhydrio-D-fructose reductase
P49429 4-hydroxyphenylpyruvate dioxygenase
P35492 Histidine ammonia-lyase
P42932 T-complex protein 1 subunit theta
Q3TLP5 Enoyl-CoA hydrtase domain-containing protein 2, mitochondrial
Q8K370 Acyl-CoA dehydrognase fmaily memember 10
O08583 THO complex subunit 4
P16331 Phenylalanine-4-hydroxylase
Q8CFA2 Aminomethyltransferase, mitochondrial
O08677 Kininogen-1
Q9QXF8 Glycine N-methyltransferase
Q61176 Arginase-1
Q9ERE7 LDLR chaperone MESD
Q91WT9 Cystathionine beta-synthase
Q91Y10 Argininosuccinate lyase
P17751 Triosephosphate isomerase
Q8CHT0 Delta-1-pyrroline-5-carboxylate dehydrogenase, mtiochondrial
P97807 Fumarate hydratase, mitochondrial
Q99KP6 Pre-mRNA-processing factor 19
P38647 Stress-70 protein, mitochondrial
P47955 60S acidic ribosomal protein P1
P16536 Spectrin alpha chain, brain
Q8BG32 26S proteasome non-ATPase regulatory subunit 11
Q9WIJA2 Phenylalainine--tRNA ligase beta subunit
P62196 26S protease regulatory subunit 8
Q8CC86 Nicotinate phosphoribosyltransferase
Q5EBG8 Uncharacterized protein C1 orf50 homoog
Q9Z1Q9 Valine--tRNA ligase
Q7TNG8 Probable D-lactate dehydrogenase, mitochondrial
P56593 Cytochrome P450 2A12
Q02248 Catenin beta-1
Q99L04 Dehydrogenase/reductase SDR family member 1
P17717 UDP-glucuronosyltransferase 2B17
Q8VC30 Bifunctional ATP-dependent dihydroxyacetone kinase/FAD-AMP lyase (cyclizing)
Q8BH86 UPFO317 protein C14orf159 homolog, mitochondrial
P47753 F-actin-capping protein subunit alpha-1
Q91VS7 Microsomal glutathione S-transferase 1
P56657 Cytochrome P450 2C40
Q9QXS1 Plectin
Q5SWU9 Acetyl-CoA carboxylase 1
Q8VCC1 15 hydroxyprostaglandin dehydrogenase [NAD+]
O88587 Catechol O-methyltransferase
Q64435 UDP-glucuronosyltransferase 1-6
Q501J6 Probable ATP-dependent RNA helicase DDX17
Q6XVG2 Cytochrome P450 2C54
P48024 Eukaryotic translation initiation factor 1
Q05421 Cytochrome P450 2E1
P20852 Cytochrome P450 2A5
P24549 Retinal dehydrogenase 1
Q8VCC2 Liver carboxylesterase 1
Q64310 Surfeit locus protein 4
P51658 Estradiol 17-beta-dehydrognease 2
Q91X77 Cytochrome P450 2C50
P53657 Pyruvate kinase isozymes R/L
Q9QXZ6 Solute carrier organic anion transporter family member 1A1
Q8VCB3 Glycogen [starch] synthase, liver
Q9ZX85 Interferon-inducible GTPase 1
Q64514 Tripeptidyl peptidase 2
Q9ET01 Glycogen phosphorylase, liver form
P52430 Serum paraoxonase/arylesterase 1
Q9DCS9 NADH dehydrogenase [ubiquinone] 1 beta subcomplex subunit 10
P99025 GTP cyclohydrolase 1 feedback regulatory protein
P35123 Ubiquitin carboxyl-terminal hydrolase 4
Q922H4 Mannoase-1-phosphate guanyltransferase alpha
Q8R0V5 Indoleamine 2,3-dioxygenase 2
Q9CQJ8 NADH dehydrogenase [ubiquinone] 1 beta subcomplex subunit 9
O35114 Lysosome membrane protein 2
P00186 Cytochrome P450 1A2
Q8BSE0 Regulator of microtubule dynamis protein 2
Q6PDI5 Proteasome-associated protein ECM29 homolog
B5X0G2 Major urinary protien 17
Q63836 Selenium-binding protein2
B
High
Low
Aged Casp2-/- VS. Aged WT
Ageing
WT
Casp2-/-
P56959 RNA-binding protein FUS
Q61694 3 beta-hydroxysteroid dehydrogenase type 5
P12265 Beta-glucuronidase
Q9R257 Heme-binding protein 1
Q9R112 Sulfide:quinone oxidoreductase, mitochondrial
P00405 Cytochrome c oxidase subunit 2
P08228 Superoxide dismutase [Cu-Zn]
Q91YP0 L-2-hydroxyglutarate dehydrogenase, mitochondrial
Q9ER88 28S ribosomal protein S29, mitochondrial
P70699 Lysosomal alpha-glucosidase
Q9WVD5 Mitochondrial ornithine transporter 1
Q8C196 Carbamoyl-phosphate synthase [ammonia], mitochondrial
Q6ZWU9 40S ribosomal protein S27
Q9DCX2 ATP synthase subunit d, mitochondrial
Q9CQQ7 ATP synthase subunit b, mitohcondrial
Q8R1I1 Cytochrome b-c1 complex subunit 9
Q922Q1 MOSC domain-contianing protein 2, mitochodnrial
Q60932 Voltage-dependent aninon-selective channel protein 1
Q64458 Cytochrome P450 2C29
Q8VEM8 Phosphate carrier protein, mitochondrial
P11714 Cytochrome P450 2D9
Q9DBW0 Cytochrome P450 4V2
Q9CQ69 Cytochrome b-c1 complex subunit 8
Q9CZ13 Cytochrome b-c1 complex subunit 1, mitochondrial
Q6XVG2 Cytochrome P450 2C54
Q8VEK3 Heterogenous nuclear ribonucleoprotien U
Q9CPQ8 ATP synthase subunit g, mitochondrial
Q4LDG0 Bile acyl-CoA synthetase
O88451 Retinol dehydrogenase 7
Q6ZQ38 Cullin-associated NEDD8-dissociated protein 1
Q91WN4 Kynurenine 3-monooxygenase
P60335 Poly(rC)-binding protien 1
P56480 ATP synthase subunit beta, mitochondrial
Q9D3D9 ATP synthase subunit delta, mitochondrial
P50544 Very long-chain specific acyl-CoA dehydrogenase, mitochondrial
Q8CFA2 Aminomethyltransferase, mitochondrial
Q99LC5 Electron transfer flavoprotein subunit alpha, mitochondrial
Q03265 ATP synthase subunit alpha, mitochondrial
Q9Z219 Succinyl-CoA ligase [ADP-forming] subunit beta, mitochondrial
P53994 Ras-related protein Rab-2A
Q922D8 C-1-tetrahydrofolate synthase, cytoplasmic
Q8CC86 Nicotinate phosphoribosyltransferase
Q571F8 Glutaminase liver isoform, mitochondrial
Q9EP89 Serine beta-lactamase-like protein LACTB, mitochondrial
Q97218 Succinyl-CoA ligase [GDP-forming] subunit beta, mitochondrial
P10126 Elongation facotr 1-alpha 1
Q9CQF9 Prenylcysteine oxidase
Q9D1Q6 Endoplasic reticulum resident protein 44
P51660 Peroxisomal multifunctional enzyme type 2
Q9QXD1 Peroxisomal acyl-coenzyme A oxidase 2
P47753 F-actin-capping protein subunit alpha-1
P45878 Peptidyl-prolyl cis-trans isomerase FKBP2
Q923D2 Flavin reducase (NADPH)
Q9DCD0 6-phosphogluconate dehydrogenase, decarboxylating
Q00897 Alpha-1-antitrypsin 1-4
Q55O60 Thiopurine S-methyltransferase
Q9WU78 Programmed cell death 6-interacting protein
Q9JLV1 BAG family molecular chaperone regulator 3
Q9QXE0 2-hydroxyacyl-CoA lyase 1
Q91VI7 Ribonuclease inhibitor
P10649 Glutathione S-transferase Mu1
Q9ROH0 Peroxisomal acyl-coenzyme A oxidase 1
P68510 14-3-3 protein beta/alpha
P0505064 Fructose-bisphosphate aldolase A
Q9D2R0 Acetyoacetyl-CoA synthetase
Q6P814 PEST proteolytic signal-containing nuclear protein
P10518 Delta-aminolevulinic acid dehydratase
Q00896 Alpha-1-atnitrypsin 13-
Q921I1 Serotransferrin
Q91XF0 Pyridoxine-5'-phoasphate oxidase
Q8VCH0 3-ketoacyl-CoA thiolase B, peroxisomal
P15626 Glutathione S-transferase Mu 2
P16045 Galectin-1
P22599 Alpha-1-antitrypsin 1-2
P51125 Calpastatin
Q9DC61 Mitochondrial-processing peptidase subunit alpha
Q91X72 Hemopexin
P07724 Serum albumin
P00920 Carbonic anhydrase 2
P01942 Hemoglobin subunit alpha
P02088 Hemoglobin subunit beta-1

Figure S1

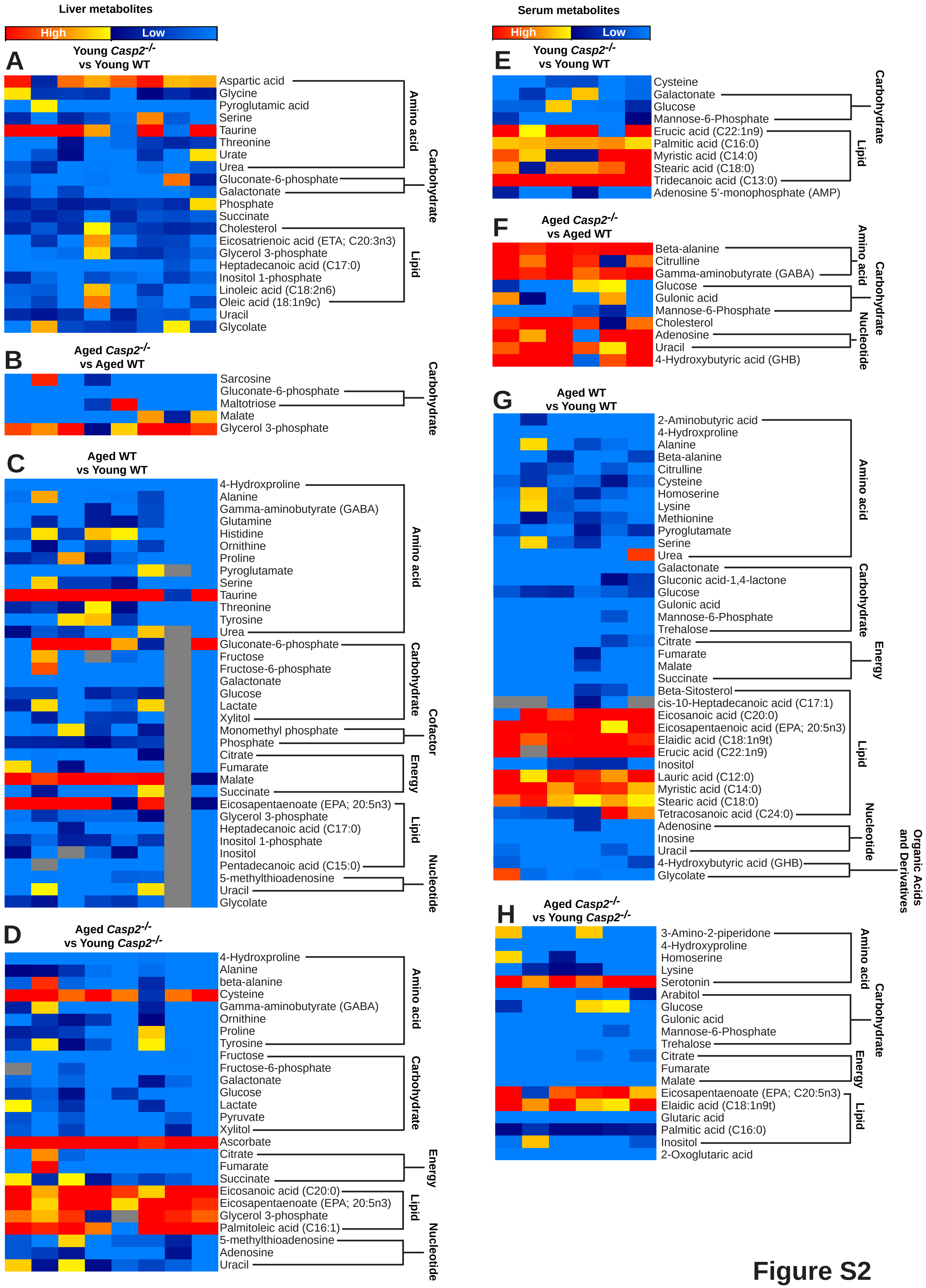

Liver metabolites
High
Low
A
Young Casp2-/- vs Young WT
Aspartic acid
Glycine
Pyroglutamic acid
Serine
Taurine
Threonine
Urate
Urea
Gluconate-6-phosphate
Galactonate
Phosphate
Succinate
Cholesterol
Eicosatrienoic acid (ETA; C20:3n3)
Glycerol 3-phosphate
Heptadecanoic acid (C17:0)
Inositol 1-phosphate
Linoleic acid (C18:2n6)
Oleic acid (18:1n9c)
Uracil
Glycolate
Amino acid
Carbohydrate
Lipid
B
Aged Casp2-/- vs Aged WT
Sarcosine
Gluconate-6-phosphate
Maltotriose
Malate
Glycerol 3-phosphate
Carbohydrate
C
Aged WT vs Young WT
4-Hydroxproline
Alanine
Gamma-aminobutyrate (GABA)
Glutamine
Histidine
Ornithine
Proline
Pyroglutamate
Serine
Taurine
Threonine
Tyrosine
Urea
Gluconate-6-phosphate
Fructose
Fructose-6-phosphate
Galactonate
Glucose
Lactate
Xylitol
Monomethyl phosphate
Phosphate
Citrate
Fumarate
Malate
Succinate
Eicosapentaenoate (EPA; 20:5n3)
Glycerol 3-phosphate
Heptadecanoic acid (C17:0)
Inositol 1-phosphate
Inositol
Pentadecanoic acid (C15:0)
5-methylthioadenosine
Uracil
Glycolate
Amino acid
Carbohydrate
Cofactor
Energy
Lipid
Nucleotide
D
Aged Casp2-/- vs Young Casp2-/-
4-Hydroxproline
Alanine
beta-alanine
Cysteine
Gamma-aminobutyrate (GABA)
Ornithine
Proline
Tyrosine
Fructose
Fructose-6-phosphate
Galactonate
Glucose
Lactate
Pyruvate
Xylitol
Ascorbate
Citrate
Fumarate
Succinate
Eicosanoic acid (C20:0)
Eicosapentaenoate (EPA; 20:5n3)
Glycerol 3-phosphate
Palmitoleic acid (C16:1)
5-methylthioadenosine
Adenosine
Uracil
Amino acid
Carbohydrate
Energy
Lipid
Nucleotide
Serum metabolites
High
Low
E
Young Casp2-/- vs Young WT
Cysteine
Galactonate
Glucose
Mannose-6-Phosphate
Erucic acid (C22:1n9)
Palmitic acid (C16:0)
Myristic acid (C14:0)
Stearic acid (C18:0)
Tridecanoic acid (C13:0)
Adenosine 5'-monophosphate (AMP)
Carbohydrate
Lipid
F
Aged Casp2-/- vs Aged WT
Beta-alanine
Citrulline
Gamma-aminobutyrate (GABA)
Glucose
Gulonic acid
Mannose-6-Phosphate
Cholesterol
Adenosine
Uracil
4-Hydroxybutyric acid (GHB)
Amino acid
Carbohydrate
Nucleotide
G
Aged WT vs Young WT
2-Aminobutyric acid
4-Hydroxproline
Alanine
Beta-alanine
Citrulline
Cysteine
Homoserine
Lysine
Methionine
Pyroglutamate
Serine
Urea
Galactonate
Gluconic acid-1,4-lactone
Glucose
Gulonic acid
Mannose-6-Phosphate
Trehalose
Citrate
Fumarate
Malate
Succinate
Beta-Sitosterol
cis-10-Heptadecanoic acid (C17:1)
Eicosanoic acid (C20:0)
Eicosapentaenoic acid (EPA; 20:5n3)
Elaidic acid (C18:1n9t)
Erucic acid (C22:1n9)
Inositol
Lauric acid (C12:0)
Myristic acid (C14:0)
Stearic acid (C18:0)
Tetracosanoic acid (C24:0)
Adenosine
Inosine
Uracil
4-Hydroxybutyric acid (GHB)
Glycolate
Amino acid
Carbohydrate
Energy
Lipid
Nucleotide
Organic Acids and Derivatives
H
Aged Casp2-/- vs Young Casp2-/-
3-Amino-2-piperidone
4-Hydroxyproline
Homoserine
Lysine
Serotonin
Arabitol
Glucose
Gulonic acid
Mannose-6-Phosphate
Trehalose
Citrate
Fumarate
Malate
Eicosapentaenoate (EPA; C20:5n3)
Elaidic acid (C18:1n9t)
Glutaric acid
Palmitic acid (C16:0)
Inositol
2-Oxoglutaric acid
Amino acid
Carbohydrate
Energy
Lipid

Figure S2

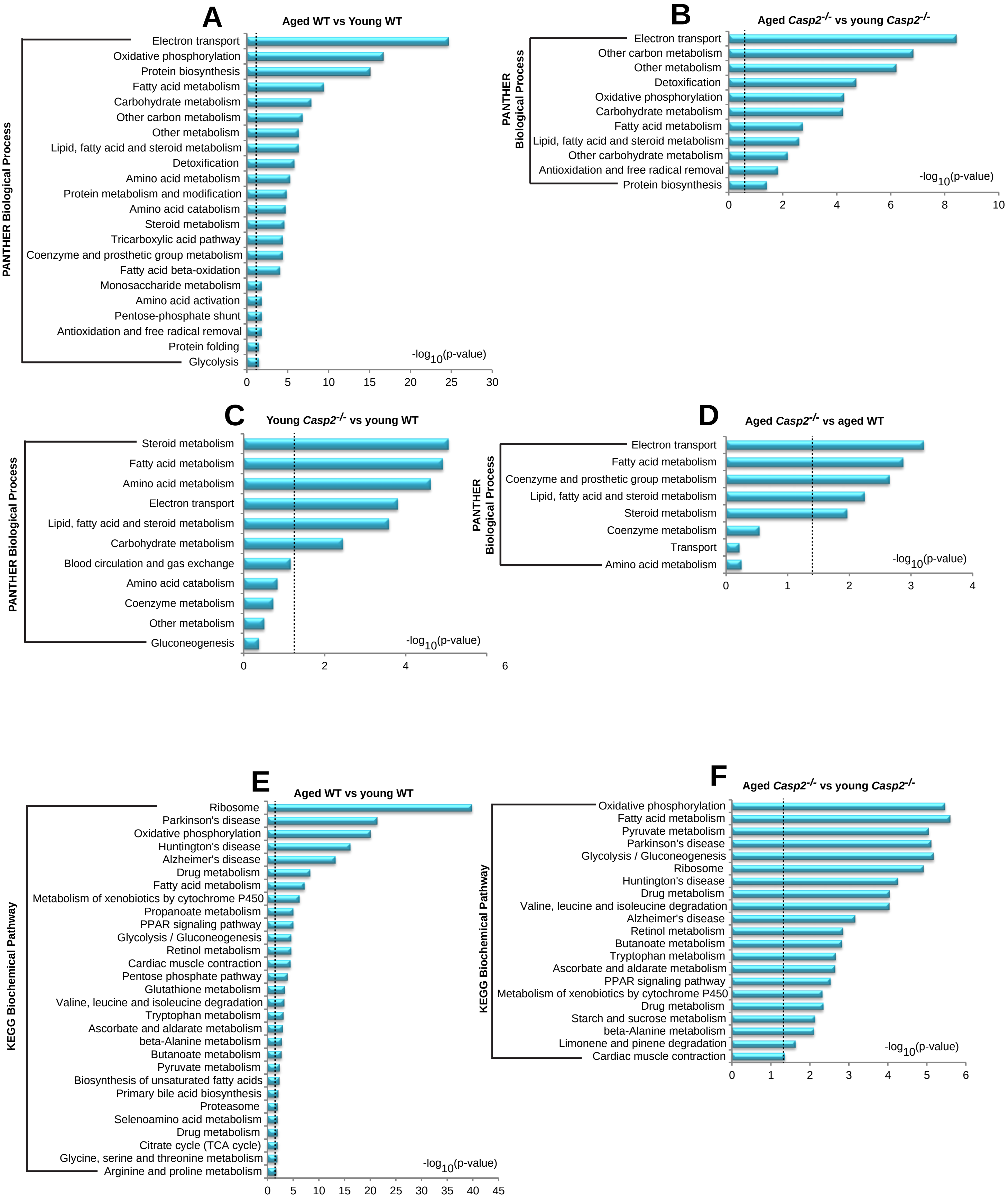

A
Aged WT vs Young WT
PANTHER Biological Process
Electron transport
Oxidative phosphorylation
Protein biosynthesis
Fatty acid metabolism
Carbohydrate metabolism
Other carbon metabolism
Other metabolism
Lipid, fatty acid and steroid metabolism
Detoxification
Amino acid metabolism
Protein metabolism and modification
Amino acid catabolism
Steroid metabolism
Tricarboxylic acid pathway
Coenzyme and prosthetic group metabolism
Fatty acid beta-oxidation
Monosaccharide metabolism
Amino acid activation
Pentose-phosphate shunt
Antioxidation and free radical removal
Protein folding
Glycolysis
-log10(p-value)
0 5 10 15 20 25 30
B
Aged Casp2-/- vs young Casp2-/-
PANTHER Biological Process
Electron transport
Other carbon metabolism
Other metabolism
Detoxification
Oxidative phosphorylation
Carbohydrate metabolism
Fatty acid metabolism
Lipid, fatty acid and steroid metabolism
Other carbohydrate metabolism
Antioxidation and free radical removal
Protein biosynthesis
-log10(p-value)
0 2 4 6 8 10
C
Young Casp2-/- vs young WT
PANTHER Biological Process
Steroid metabolism
Fatty acid metabolism
Amino acid metabolism
Electron transport
Lipid, fatty acid and steroid metabolism
Carbohydrate metabolism
Blood circulation and gas exchange
Amino acid catabolism
Coenzyme metabolism
Other metabolism
Gluconeogenesis
-log10(p-value)
0 2 4 6
D
Aged Casp2-/- vs aged WT
PANTHER Biological Process
Electron transport
Fatty acid metabolism
Coenzyme and prosthetic group metabolism
Lipid, fatty acid and steroid metabolism
Steroid metabolism
Coenzyme metabolism
Transport
Amino acid metabolism
-log10(p-value)
0 1 2 3 4
E
Aged WT vs young WT
KEGG Biochemical Pathway
Ribosome
Parkinson's disease
Oxidative phosphorylation
Huntington's disease
Alzheimer's disease
Drug metabolism
Fatty acid metabolism
Metabolism of xenobiotics by cytochrome P450
Propanoate metabolism
PPAR signaling pathway
Glycolysis / Gluconeogenesis
Retinol metabolism
Cardiac muscle contraction
Pentose phosphate pathway
Glutathione metabolism
Valine, leucine and isoleucine degradation
Tryptophan metabolism
Ascorbate and aldarate metabolism
beta-Alanine metabolism
Butanoate metabolism
Pyruvate metabolism
Biosynthesis of unsaturated fatty acids
Primary bile acid biosynthesis
Proteasome
Selenoamino acid metabolism
Drug metabolism
Citrate cycle (TCA cycle)
Glycine, serine and threonine metabolism
Arginine and proline metabolism
-log10(p-value)
0 5 10 15 20 25 30 35 40 45
F
Aged Casp2-/- vs young Casp2-/-
KEGG Biochemical Pathway
Oxidative phosphorylation
Fatty acid metabolism
Pyruvate metabolism
Parkinson's disease
Glycolysis / Gluconeogenesis
Ribosome
Huntington's disease
Drug metabolism
Valine, leucine and isoleucine degradation
Alzheimer's disease
Retinol metabolism
Butanoate metabolism
Tryptophan metabolism
Ascorbate and aldarate metabolism
PPAR signaling pathway
Metabolism of xenobiotics by cytochrome P450
Drug metabolism
Starch and sucrose metabolism
beta-Alanine metabolism
Limonene and pinene degradation
Cardiac muscle contraction
-log10(p-value)
0 1 2 3 4 5 6

Figure S3

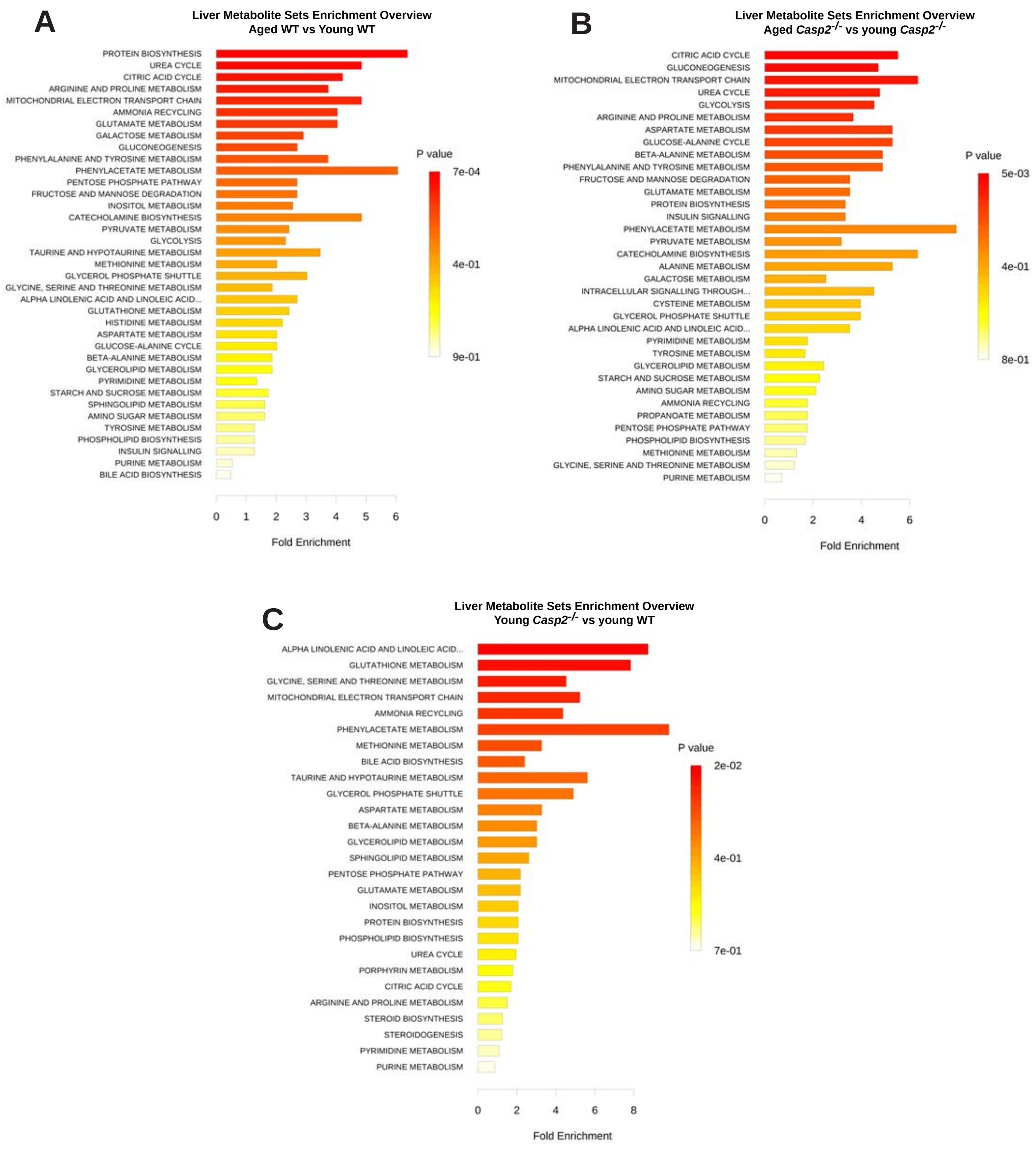
A
Liver Metabolite Sets Enrichment Overview
Aged WT vs Young WT
PROTEIN BIOSYNTHESIS
UREA CYCLE
CITRIC ACID CYCLE
ARGININE AND PROLINE METABOLISM
MITOCHONDRIAL ELECTRON TRANSPORT CHAIN
AMMONIA RECYCLING
GLUTAMATE METABOLISM
GALACTOSE METABOLISM
GLUCONEOGENESIS
PHENYLALANINE AND TYROSINE METABOLISM
PHENYLACETATE METABOLISM
PENTOSE PHOSPHATE PATHWAY
FRUCTOSE AND MANNOSE DEGRADATION
INOSITOL METABOLISM
CATECHOLAMINE BIOSYNTHESIS
PYRUVATE METABOLISM
GLYCOLYSIS
TAURINE AND HYPOTAURINE METABOLISM
METHIONINE METABOLISM
GLYCEROL PHOSPHATE SHUTTLE
GLYCINE, SERINE AND THREONINE METABOLISM
ALPHA LINOLENIC ACID AND LINOLEIC ACID...
GLUTATHIONE METABOLISM
HISTIDINE METABOLISM
ASPARTATE METABOLISM
GLUCOSE-ALANINE CYCLE
BETA-ALANINE METABOLISM
GLYCEROLIPID METABOLISM
PYRIMIDINE METABOLISM
STARCH AND SUCROSE METABOLISM
SPHINGOLIPID METABOLISM
AMINO SUGAR METABOLISM
TYROSINE METABOLISM
PHOSPHOLIPID BIOSYNTHESIS
INSULIN SIGNALLING
PURINE METABOLISM
BILE ACID BIOSYNTHESIS
P value
7e-04
4e-01
9e-01
0 1 2 3 4 5 6
Fold Enrichment
B
Liver Metabolite Sets Enrichment Overview
Aged Casp2-/- vs young Casp2-/-
CITRIC ACID CYCLE
GLUCONEOGENESIS
MITOCHONDRIAL ELECTRON TRANSPORT CHAIN
UREA CYCLE
GLYCOLYSIS
ARGININE AND PROLINE METABOLISM
ASPARTATE METABOLISM
GLUCOSE-ALANINE CYCLE
BETA-ALANINE METABOLISM
PHENYLALANINE AND TYROSINE METABOLISM
FRUCTOSE AND MANNOSE DEGRADATION
GLUTAMATE METABOLISM
PROTEIN BIOSYNTHESIS
INSULIN SIGNALLING
PHENYLACETATE METABOLISM
PYRUVATE METABOLISM
CATECHOLAMINE BIOSYNTHESIS
ALANINE METABOLISM
GALACTOSE METABOLISM
INTRACELLULAR SIGNALLING THROUGH...
CYSTEINE METABOLISM
GLYCEROL PHOSPHATE SHUTTLE
ALPHA LINOLENIC ACID AND LINOLEIC ACID...
PYRIMIDINE METABOLISM
TYROSINE METABOLISM
GLYCEROLIPID METABOLISM
STARCH AND SUCROSE METABOLISM
AMINO SUGAR METABOLISM
AMMONIA RECYCLING
PROPANOATE METABOLISM
PENTOSE PHOSPHATE PATHWAY
PHOSPHOLIPID BIOSYNTHESIS
METHIONINE METABOLISM
GLYCINE, SERINE AND THREONINE METABOLISM
PURINE METABOLISM
P value
5e-03
4e-01
8e-01
0 2 4 6
Fold Enrichment
C
Liver Metabolite Sets Enrichment Overview
Young Casp2-/- vs young WT
ALPHA LINOLENIC ACID AND LINOLEIC ACID...
GLUTATHIONE METABOLISM
GLYCINE, SERINE AND THREONINE METABOLISM
MITOCHONDRIAL ELECTRON TRANSPORT CHAIN
AMMONIA RECYCLING
PHENYLACETATE METABOLISM
METHIONINE METABOLISM
BILE ACID BIOSYNTHESIS
TAURINE AND HYPOTAURINE METABOLISM
GLYCEROL PHOSPHATE SHUTTLE
ASPARTATE METABOLISM
BETA-ALANINE METABOLISM
GLYCEROLIPID METABOLISM
SPHINGOLIPID METABOLISM
PENTOSE PHOSPHATE PATHWAY
GLUTAMATE METABOLISM
INOSITOL METABOLISM
PROTEIN BIOSYNTHESIS
PHOSPHOLIPID BIOSYNTHESIS
UREA CYCLE
PORPHYRIN METABOLISM
CITRIC ACID CYCLE
ARGININE AND PROLINE METABOLISM
STEROID BIOSYNTHESIS
STEROIDOGENESIS
PYRIMIDINE METABOLISM
PURINE METABOLISM
P value
2e-02
4e-01
7e-01
0 2 4 6 8
Fold Enrichment

Figure S4

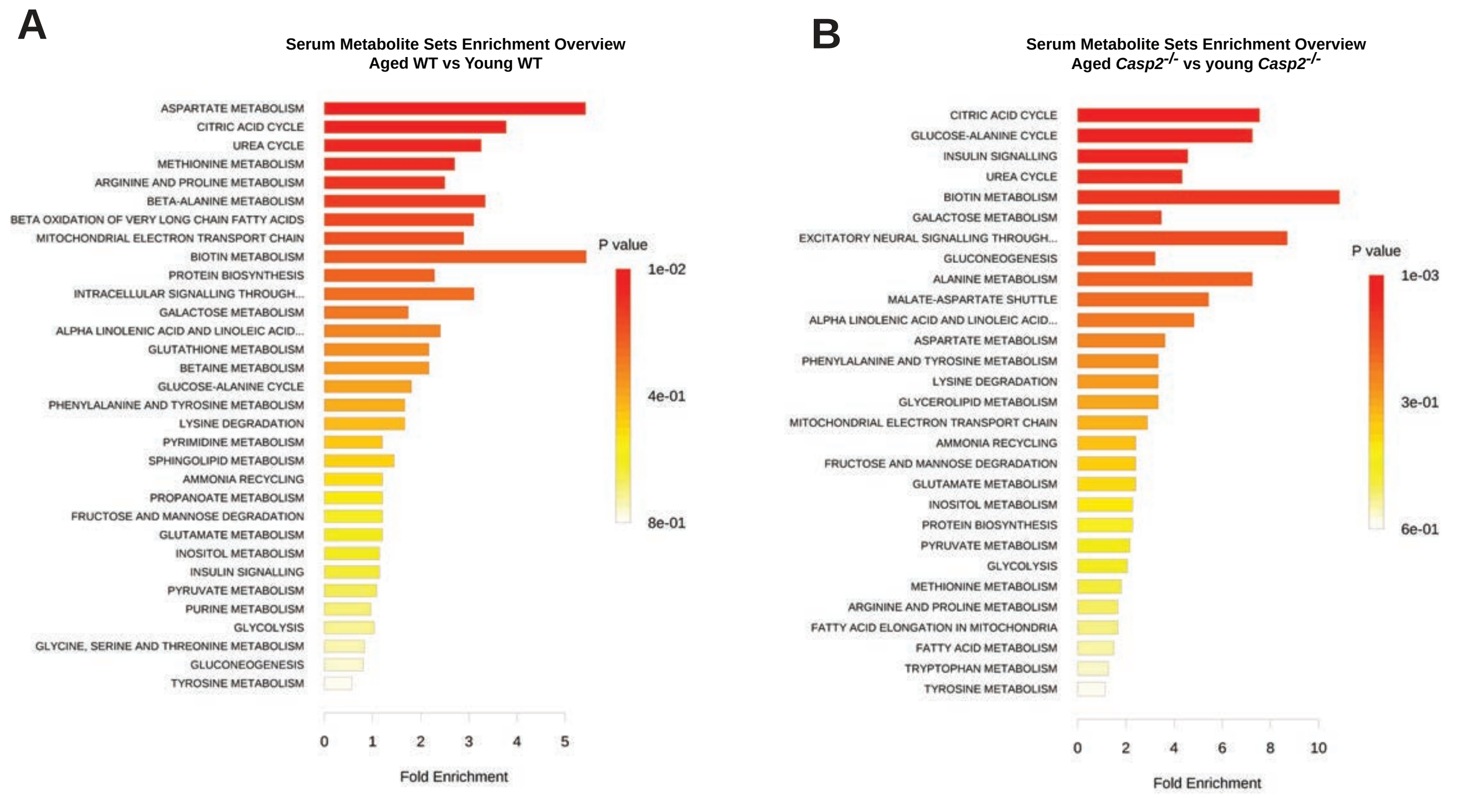
A
Serum Metabolite Sets Enrichment Overview
Aged WT vs Young WT
ASPARTATE METABOLISM
CITRIC ACID CYCLE
UREA CYCLE
METHIONINE METABOLISM
ARGININE AND PROLINE METABOLISM
BETA-ALANINE METABOLISM
BETA OXIDATION OF VERY LONG CHAIN FATTY ACIDS
MITOCHONDRIAL ELECTRON TRANSPORT CHAIN
BIOTIN METABOLISM
PROTEIN BIOSYNTHESIS
INTRACELLULAR SIGNALLING THROUGH...
GALACTOSE METABOLISM
ALPHA LINOLENIC ACID AND LINOLEIC ACID...
GLUTATHIONE METABOLISM
BETAINE METABOLISM
GLUCOSE-ALANINE CYCLE
PHENYLALANINE AND TYROSINE METABOLISM
LYSINE DEGRADATION
PYRIMIDINE METABOLISM
SPHINGOLIPID METABOLISM
AMMONIA RECYCLING
PROPANOATE METABOLISM
FRUCTOSE AND MANNOSE DEGRADATION
GLUTAMATE METABOLISM
INOSITOL METABOLISM
INSULIN SIGNALLING
PYRUVATE METABOLISM
PURINE METABOLISM
GLYCOLYSIS
GLYCINE, SERINE AND THREONINE METABOLISM
GLUCONEOGENESIS
TYROSINE METABOLISM
0
1
2
3
4
5
Fold Enrichment
P value
1e-02
4e-01
8e-01
B
Serum Metabolite Sets Enrichment Overview
Aged *Casp2*$^{-/-}$ vs young *Casp2*$^{-/-}$
CITRIC ACID CYCLE
GLUCOSE-ALANINE CYCLE
INSULIN SIGNALLING
UREA CYCLE
BIOTIN METABOLISM
GALACTOSE METABOLISM
EXCITATORY NEURAL SIGNALLING THROUGH...
GLUCONEOGENESIS
ALANINE METABOLISM
MALATE-ASPARTATE SHUTTLE
ALPHA LINOLENIC ACID AND LINOLEIC ACID...
ASPARTATE METABOLISM
PHENYLALANINE AND TYROSINE METABOLISM
LYSINE DEGRADATION
GLYCEROLIPID METABOLISM
MITOCHONDRIAL ELECTRON TRANSPORT CHAIN
AMMONIA RECYCLING
FRUCTOSE AND MANNOSE DEGRADATION
GLUTAMATE METABOLISM
INOSITOL METABOLISM
PROTEIN BIOSYNTHESIS
PYRUVATE METABOLISM
GLYCOLYSIS
METHIONINE METABOLISM
ARGININE AND PROLINE METABOLISM
FATTY ACID ELONGATION IN MITOCHONDRIA
FATTY ACID METABOLISM
TRYPTOPHAN METABOLISM
TYROSINE METABOLISM
0
2
4
6
8
10
Fold Enrichment
P value
1e-03
3e-01
6e-01

Figure S5

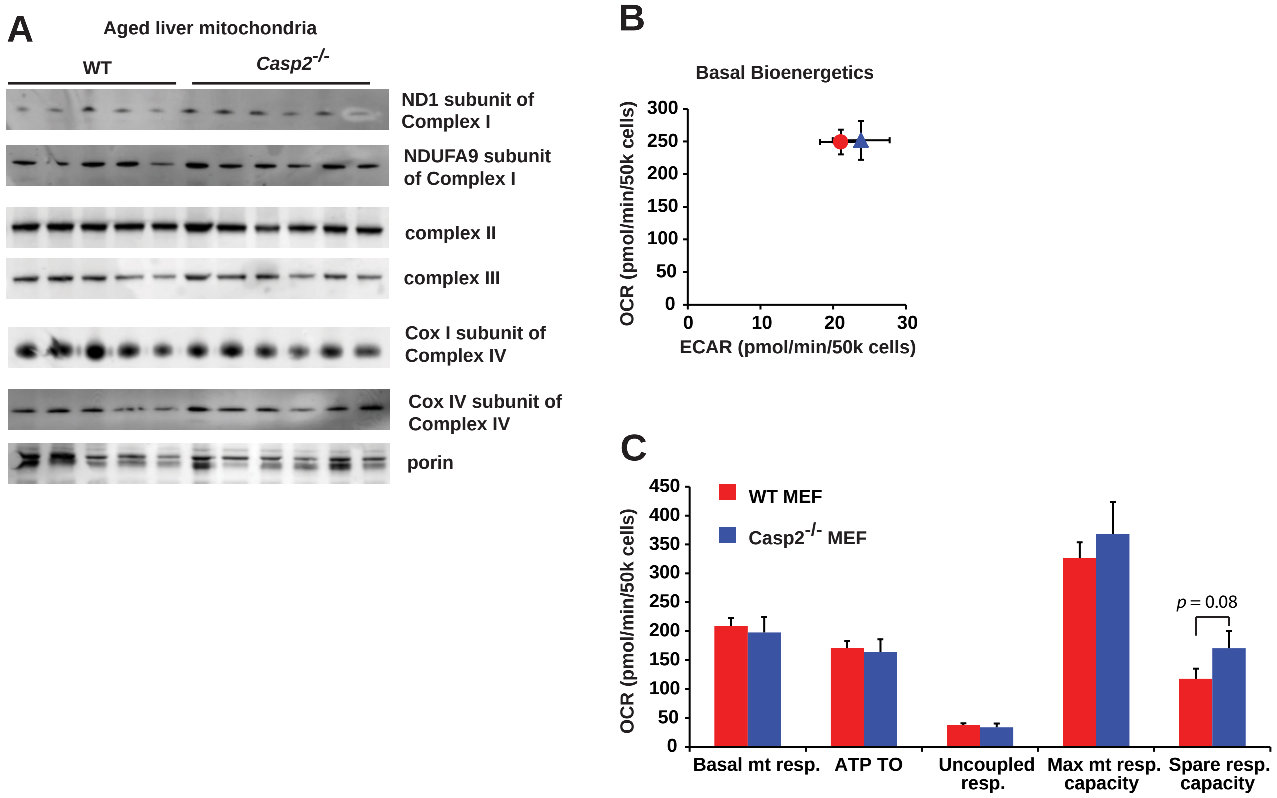
A
Aged liver mitochondria
WT
Casp2-/-
ND1 subunit of Complex I
NDUFA9 subunit of Complex I
complex II
complex III
Cox I subunit of Complex IV
Cox IV subunit of Complex IV
porin
B
Basal Bioenergetics
OCR (pmol/min/50k cells)
ECAR (pmol/min/50k cells)
C
WT MEF
Casp2-/- MEF
OCR (pmol/min/50k cells)
p = 0.08
Basal mt resp.
ATP TO
Uncoupled resp.
Max mt resp. capacity
Spare resp. capacity

Figure S6
